# The Computational Anatomy of Psychosis

**DOI:** 10.3389/fpsyt.2013.00047

**Published:** 2013-05-30

**Authors:** Rick A. Adams, Klaas Enno Stephan, Harriet R. Brown, Christopher D. Frith, Karl J. Friston

**Affiliations:** ^1^Wellcome Trust Centre for Neuroimaging, Institute of Neurology, University College London, London, UK.; ^2^Translational Neuromodeling Unit, Institute for Biomedical Engineering, University of Zurich, ETH Zurich, Zurich, Switzerland; ^3^Laboratory for Social and Neural Systems Research, University of Zurich, Zurich, Switzerland

**Keywords:** free energy, active inference, precision, sensory attenuation, illusions, psychosis, schizophrenia

## Abstract

This paper considers psychotic symptoms in terms of false inferences or beliefs. It is based on the notion that the brain is an inference machine that actively constructs hypotheses to explain or predict its sensations. This perspective provides a normative (Bayes-optimal) account of action and perception that emphasizes probabilistic representations; in particular, the confidence or *precision* of beliefs about the world. We will consider hallucinosis, abnormal eye movements, sensory attenuation deficits, catatonia, and delusions as various expressions of the same core pathology: namely, an aberrant encoding of precision. From a cognitive perspective, this represents a pernicious failure of metacognition (beliefs about beliefs) that can confound perceptual inference. In the embodied setting of active (Bayesian) inference, it can lead to behaviors that are paradoxically more accurate than Bayes-optimal behavior. Crucially, this normative account is accompanied by a neuronally plausible process theory based upon hierarchical predictive coding. In predictive coding, precision is thought to be encoded by the post-synaptic gain of neurons reporting prediction error. This suggests that both pervasive *trait* abnormalities and florid failures of inference in the psychotic *state* can be linked to factors controlling post-synaptic gain – such as NMDA receptor function and (dopaminergic) neuromodulation. We illustrate these points using biologically plausible simulations of perceptual synthesis, smooth pursuit eye movements and attribution of agency – that all use the same predictive coding scheme and pathology: namely, a reduction in the precision of prior beliefs, relative to sensory evidence.

## Introduction

This paper attempts to explain the positive and negative symptoms of schizophrenia in terms of false inference about states of the world producing sensations – and to link this explanation to neuromodulatory dysconnections at the synaptic level. In brief, we take a normative approach to action and perception – namely, active inference and the Bayesian brain hypothesis. We then consider neuronally plausible implementations of active inference to see how particular failures of neuromodulation would be expressed in terms of perceptual inference and behavior. The main conclusion is that a wide range of psychotic symptoms can be explained by a failure to represent the precision of beliefs about the world – and that this failure corresponds to abnormal neuromodulation of the post-synaptic gain of superficial pyramidal cells in cortical hierarchies. This may sound like a very specific assertion; however, there are many converging lines of evidence that point to this conclusion – lines that we try to draw together in this paper.

The basic idea is that faulty inference leads to false concepts (delusions) or percepts (hallucinations) and that this failure is due to a misallocation of precision to hierarchical representations in the brain. In what follows, we will refer to beliefs, inference, priors, and precision in a Bayesian sense. In this setting, a *belief* is a probability distribution over some unknown state or attribute. Beliefs, in this sense, may or may not be consciously accessible. A belief can be held with great *precision*, such that the probability distribution is concentrated over the most likely value – the mean or *expectation*. This means the precision (inverse variance) corresponds to the confidence or certainty associated with a belief. In Bayesian inference, beliefs prior to observing data are called *prior beliefs*, which are updated to *posterior beliefs* after seeing the data. This updating rests upon combining a prior belief with sensory evidence or the *likelihood* of the data. In hierarchical Bayesian inference, the *sufficient statistics* of a belief (like the expectation and precision) are themselves treated as unknown quantities. This means that one can have beliefs about beliefs; for example, one can have an expectation about a precision (c.f., expected uncertainty). Heuristically, this leads to the distinction between fixed and random effects in classical statistics; or between risk (known uncertainty) and ambiguity (unknown uncertainty) in economics. Beliefs about beliefs are inevitable in hierarchical inference and are sometimes referred to as *empirical priors*, because they provide constraints on beliefs at lower levels of the hierarchy. Behaviorally, precision and beliefs about precision (including subjective confidence in beliefs) are to some extent dissociable (Fleming et al., 2012). Beliefs about precision are particularly important in hierarchical Bayesian inference, because they can have a profound effect on posterior expectations – and inappropriate beliefs about precision can easily lead to false inference.

**Figure 1:**
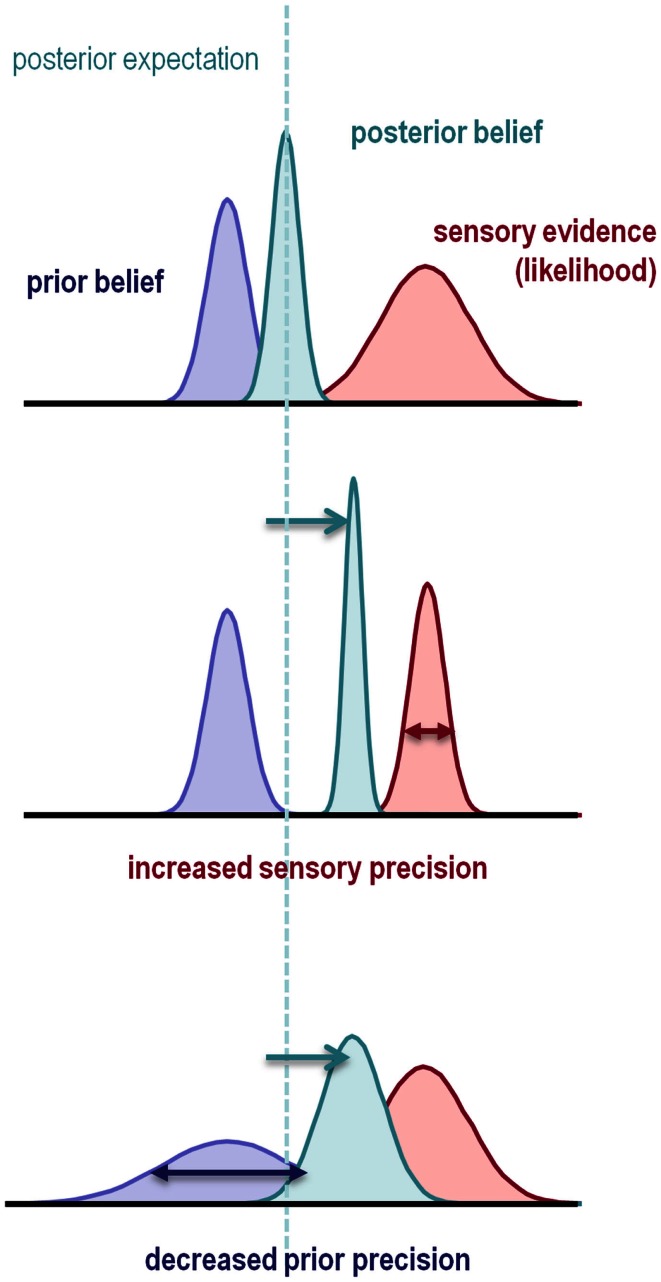
**This schematic illustrates the importance of precision when forming posterior beliefs and expectations**. The graphs show Gaussian probability distributions that represent prior beliefs, posterior beliefs, and the likelihood of some data or sensory evidence as functions of some hidden (unknown) parameter. The dotted line corresponds to the posterior expectation, while the width of the distributions corresponds to their dispersion or variance. Precision is the inverse of this dispersion and can have a profound effect on posterior beliefs. Put simply, the posterior belief is biased toward the prior or sensory evidence in proportion to their relative precision. This means that the posterior expectation can be biased toward sensory evidence by either increasing sensory precision – or failing to attenuate it – or by decreasing prior precision.

The nature of this failure can be understood intuitively by considering classical statistical inference: imagine that we are using a *t*-test to compare the mean of some data, against the null hypothesis that the mean is zero. The sample mean provides evidence against the null hypothesis in the form of a *prediction error*: namely, the sample mean minus the expectation under the null hypothesis. The sample mean provides evidence against the null but how much evidence? This can only be quantified in relation to the precision of the prediction error. The *t*-statistic is simply the prediction error weighted by its precision (i.e., divided by its standard error). If this precision-weighted prediction error is sufficiently large, one rejects the null hypothesis. Clearly, if we overestimate the precision of the data, the *t*-statistic will be too large and we expose ourselves to false positives. Analogous rules apply to Bayesian inference, in that the optimal combination of a prior belief with some evidence is a posterior belief whose mean is a mixture of the prior and data means, weighted according to their precision. If the precision of the data is overestimated, or if the precision of the prior is underestimated, the posterior expectation will shift from the prior mean to the data mean (Figure 1).

So how could this lead to false beliefs and delusions? The following scenario (Frith and Friston, 2012) illustrates this: imagine the temperature warning light in your car is too sensitive (precise), reporting the slightest fluctuations (prediction errors) above some temperature. You naturally infer that there is something wrong with your car and take it to the garage. However, they find no fault – and yet the warning light continues to flash. Your first instinct may be to suspect the garage has failed to identify the fault – and even to start to question the Good Garage Guide that recommended it. From your point of view, these are all plausible hypotheses that accommodate the evidence available to you. However, from the perspective of somebody who has never seen your warning light, your suspicions would have an irrational and slightly paranoid flavor. This anecdote illustrates how delusional systems may be elaborated as a consequence of imbuing sensory evidence with too much precision. Note that the primary pathology here is quintessentially metacognitive in nature: in the sense that it rests on a belief (the warning light reports precise information) about a belief (the engine is overheating). Crucially, there is no necessary impairment in forming predictions or prediction errors – the problem lies in the way they are used to inform inference or hypotheses.

In what follows, we will consider the brain as performing inference using predictive coding, in which the evidence for hypotheses is reported by precision-weighted prediction errors. In these schemes, certain neurons compare bottom-up inputs with top-down predictions to form a prediction error that is weighted in proportion to its expected precision. Crucially, this weighting corresponds to the gain or sensitivity of prediction error units. This means that abnormalities in the modulation of post-synaptic gain could, in principle, lead to false inferences of the sort described above. We will illustrate this in a concrete fashion using biologically plausible simulations of false inference, all of which use exactly the same predictive coding scheme and intervention; namely, a decrease in the precision (post-synaptic gain of prediction error units) at higher levels of cortical hierarchies, relative to the precision at sensory levels. Some of these simulations have been reported previously in different contexts (Friston and Kiebel, 2009a; Adams et al., 2012; Brown et al., in press). Here, we frame these simulations in terms of false inference and emphasize their common mechanisms. There are several other examples that we could have used; for example, the relationship between state-dependent precision and attention or the role of dopamine in encoding the precision of affordance and its effects on action selection. However, the examples chosen are sufficient to illustrate the diverse phenomenology that can be explained by one simple abnormality – a reduction in the precision of empirical prior beliefs, relative to sensory precision.

This paper focuses on false inference. However, the normative principles we appeal to cover both *inference* and *learning*. Neurobiologically, this corresponds to the distinction between updating neuronal representations in terms of synaptic activity and learning causal structure through updating synaptic efficacy (i.e., synaptic plasticity). The important thing here is that abnormal beliefs about precision also lead to *false learning*, which produces – and is produced by – false inference. This circular causality follows inevitably from the nature of inference, which induces posterior dependencies among estimates of hidden quantities in the world (encoded by synaptic activity and efficacy respectively). The point here is that a simple failure of neuromodulation (and implicit encoding of precision) can have far-reaching and knock-on effects that can be manifest at many different levels of perceptual inference, learning, and consequent behavior.

This paper comprises six sections. We start with a brief review of the symptoms and signs of schizophrenia, with a special focus on how *trait* and *state* abnormalities can be cast in terms of false inference. The second section reviews the psychopharmacology of psychosis with an emphasis on the synaptic (neuromodulatory) mechanisms that we suppose underlie false inference. The third establishes the normative theory (active inference) and its biological instantiation in the brain (generalized Bayesian filtering or predictive coding). The resulting scheme is used in the final three sections to illustrate failures of perceptual inference in the context of omission paradigms, abnormalities of active inference in the context of smooth pursuit eye movements and misattribution of agency in the context of deficits in sensory attenuation.

## Psychosis and False Inference

In this section, we briefly review the state and trait abnormalities of schizophrenia to emphasize a common theme; namely, a failure of inference about the world that arises from an imbalance in the precision or confidence attributed to beliefs. We distinguish between state and trait abnormalities because the evidence suggests that trait abnormalities may be associated with a relative decrease in prior precision, while some state abnormalities can be explained by a (possibly compensatory) increase in prior precision (or reduction in sensory precision). In this setting, state abnormalities include the florid (Schneiderian or first rank) symptoms of acute psychosis, while trait abnormalities are more pervasive and subtle. The diagnostic criteria for schizophrenia are based largely on state abnormalities, because they are easily and reliably detected. These include:

- Delusions and hallucinations: c.f., positive symptoms (Crow, 1980) and the reality distortion of chronic schizophrenia (Liddle, 1987).
- Thought disorder and catatonia (World Health Organization, 1992; American Psychiatric Association, 2000), where formal thought disorder is also characteristic of the disorganization syndrome of chronic schizophrenia (Liddle, 1987). Other (as yet non-diagnostic) state abnormalities include:
- Abnormalities of perceptual organization: in particular a decreased influence of context, leading to a loss of global (Gestalt) organization (Phillips and Silverstein, 2003). These abnormalities have not been found in first-degree relatives or before the first psychotic episode, and tend to covary with disorganization symptoms (reviewed in Silverstein and Keane, 2011). A decreased influence of context can sometimes lead to perceptions that are more veridical than those of normal subjects. Important examples here include a resistance to the hollow mask illusion – which is also state-dependent (Keane et al., in press) – and size-weight illusion (Williams et al., 2010).

These symptoms can occur episodically and – with the possible exception of catatonia-respond well to anti-dopaminergic drugs in the majority of patients. We use the term “trait” abnormalities to refer to more constant features of the disorder, which are less responsive to dopamine blockade (although these responses have not been explored as thoroughly as those of state symptoms). Some are found in first-degree relatives and high-risk groups, and may qualify as endophenotypes of schizophrenia. Despite their prevalence, they are less diagnostic because they are found in other diagnostic categories (and to some extent in the normal population). They include (among others):

- Soft neurological signs: probably best exemplified by abnormalities of smooth pursuit eye movements (SPEM) as reviewed by O’Driscoll and Callahan (2008). These abnormalities are present in first-degree relatives (Calkins et al., 2008) and in drug naive first episode schizophrenics (Campion et al., 1992; Sweeney et al., 1994; Hutton et al., 1998), and may even be exacerbated by dopamine blockade (Hutton et al., 2001).
- Abnormal event-related potentials: such as a larger P50 response to a repeated stimulus, and reduced P300 and mismatch negativity (MMN) responses to violations or oddball stimuli. Abnormal P50, P300, and MMN responses have also been demonstrated in first-degree relatives, and do not normalize with treatment (reviewed in Winterer and McCarley, 2011).
- Anhedonia, cognitive impairments, and negative symptoms: such as loss of normal affect, experience of pleasure, motivation, and sociability are all found (subclinically) in first-degree relatives (Fanous et al., 2001; Jabben et al., 2010) to a greater or lesser degree (Johnstone et al., 1987; Mockler et al., 1997) and are notoriously resistant to anti-dopaminergic treatment.

Many trait abnormalities have been considered as the result of a failure to adequately predict sensory input, rendering all percepts surprising (e.g., the P50) and reducing differential responses to oddball stimuli (e.g., the MMN and P300). Predictive coding in particular has been used in recent formulations of these deficits in schizophrenia (Fletcher and Frith, 2009). Specifically, it is suggested that the main problem in schizophrenia lies not with the prediction of sensory input *per se*, but in the delicate balance of precision ascribed to prior beliefs and sensory evidence (Friston, 2005; Corlett et al., 2011). Later, we will use simulations to demonstrate how a relative increase in – or failure to attenuate – sensory precision can explain abnormal responses to surprising events.

In terms of cognitive paradigms, the “beads task” has been used to characterize formal beliefs and probabilistic reasoning in schizophrenic subjects. In this paradigm, subjects are told that red and green beads are drawn at random from an urn that contains (for example) 85% of one color and 15% of the other. The subject must decide which color predominates. In reality, all subjects are shown the same sequence of beads. In the *draws to decision* version of the task, the subject has to answer as soon as they are certain. In the *probability estimates* version, the subject can continue to draw and change their answer. Interestingly, delusional patients “jump to conclusions” in the first version, while they are more willing to revise their decision in light of contradictory evidence in the second (Garety and Freeman, 1999). Bayesian modeling suggests that jumping to conclusions may reflect greater “cognitive noise” in delusional patients (Moutoussis et al., 2011), which may speak to reduced precision of higher level (cognitive) representations and consequently a greater influence of new sensory evidence (Speechley et al., 2010).

Can state abnormalities also be explained by imbalances in the precisions of prior beliefs and sensations? The short answer is yes. For example, delusional mood describes a state in which patients feel the world is strange and has changed in some way – where their attention is drawn to apparently irrelevant stimuli and odd coincidences. A loss of precise prior beliefs is consistent with a sense of unpredictability and greater attention to sensory events. Indeed, this line of thinking has been used to explain the loss of Gestalt or central coherence in autism (Pellicano and Burr, 2012). In terms of formal models, the top-down control of sensory precision has been shown to explain several psychophysical and physiological aspects of attention (Feldman and Friston, 2010); thereby providing a formal link between precision and attention. The key insight from these models is that posterior beliefs about states of the world can direct attention to sensory features by top-down modulation of sensory precision. A failure of top-down attenuation of sensory precision (sensory attenuation) therefore fits comfortably with abnormalities of sensory attention in this context.

State abnormalities include the cardinal psychotic symptoms, such as hallucinations and delusions. Hallucinations could be understood as the result of an *increase* in the relative precision of prior beliefs, such that the posterior beliefs are impervious to contradictory – but imprecise – sensory evidence. This has been discussed as an explanation for visual hallucinosis in organic psychosyndromes (Friston, 2005). However, the hallucinations associated with psychosis may be better understood as a failure to attenuate the sensory consequences (corollary discharge) of self-made acts; for example, a failure to attenuate the auditory consequences of sub-vocal or inner speech (Frith et al., 1998; Allen et al., 2007). Delusions are probably more complex and their emergence may be better understood as secondary phenomena: several authors have proposed that they could arise as rational (Bayes-optimal) posterior beliefs that explain away precise sensory prediction errors: e.g., Fletcher and Frith (2009). These explanations relate to earlier “empiricist” accounts such as Maher (1974), Gray et al. (1991), and Kapur (2003), who emphasizes aberrant salience (c.f., sensory precision). Implicit in these secondary accounts is a compensatory increase in the precision of explanations for sensory cues that are imbued with too much precision or salience. This is consistent with their peculiar resistance to rational argument. In the final section, we will consider an example of a compensatory increase in the precision of high-level beliefs that is necessary to compensate for a failure of sensory attenuation.

### Summary

In summary, the symptoms and signs of schizophrenia are not inconsistent with a reduction of high-level precision or a failure of sensory attenuation (the top-down attenuation of sensory precision), with compensatory (secondary) changes in the precision of (empirical) prior beliefs. In particular, some psychotic states may reflect a compensatory response to trait abnormalities that bias inference toward sensory evidence that is imbued with too much precision or salience. A further mechanistic dissociation between state and trait abnormalities is suggested by the fact that the former generally respond to antipsychotic (anti-dopaminergic) treatment, while trait abnormalities do not. Before considering the computational anatomy of hierarchical inference in the brain, we will briefly review the psychopharmacology and neuropathology of schizophrenia.

## The Psychopharmacology of Precision

This section considers the neuromodulatory processes implicated in schizophrenia, with a special focus on the laminar specificity of cortical neuromodulation. Our premise here is that psychotic abnormalities are manifestations of false inference, caused by the aberrant encoding of precision. This precision is thought to be encoded by post-synaptic gain of neuronal populations reporting prediction errors – the principal or pyramidal cells of superficial cortical layers (Mumford, 1992; Feldman and Friston, 2010). Synaptic gain modulation is a change in the response amplitude of a neuron that is independent of its selectivity or receptive field characteristics (Salinas and Thier, 2000). In other words, post-synaptic gain is a factor that quantifies the effect of a presynaptic input on post-synaptic output (e.g., depolarization at the soma). Changes in synaptic gain are generally thought to be mediated by non-linear (e.g., multiplicative) synaptic mechanisms; for example, NMDA receptor activation.

Of all the receptors that determine synaptic gain, the most ubiquitous is the glutamatergic NMDA receptor (NMDA-R). NMDA-Rs have several important functions that are expressed over different timescales. First, they can drive (i.e., induce an excitatory post-synaptic potential) post-synaptic cells like other ionotropic glutamatergic (AMPA and Kainate) receptors. However, the driving effect of NMDA-Rs is only possible if the cell is already depolarized; otherwise, the NMDA-R is blocked by a magnesium ion. This non-linear property makes them synaptic coincidence detectors or “AND gates.” Second, NMDA-Rs have time constants that are much longer than that of AMPA-Rs and Kainate-Rs. This enables integration of synaptic inputs over tens to hundreds of milliseconds – increasing the gain of synaptic inputs to distal dendrites. Finally, NMDA-Rs are famous for their role in plasticity: at longer timescales, the influx of calcium ions through NMDA-R channels causes a cascade of intracellular events that result in long-term synaptic depression or potentiation (LTD or LTP). However, NMDA-Rs also have a major impact on the short-term plasticity of glutamatergic synapses. This is because they regulate the functional state and number of AMPA-Rs – by phosphorylation or by changing the trafficking of AMPA-R subunits to and from the cell membrane (Passafaro et al., 2001; Montgomery and Madison, 2004; Bagal et al., 2005). Together, these properties make a significant contribution to the dynamics of neural networks, especially to oscillatory behavior and sustained firing patterns (Durstewitz, 2009).

Other key determinants of synaptic gain are the classical neuromodulator receptors; e.g., dopamine (DA-Rs), acetylcholine (in particular muscarinic AChRs), and serotonin (5-HTRs). With the exception of nicotinic AChRs (which are ionotropic) these are all metabotropic receptors – they do not activate ion channels but are coupled to signal transduction mechanisms (via G proteins) that affect intracellular second messengers, such as cyclic adenosine monophosphate (cAMP) or cyclic guanosine monophosphate (cGMP). Fluctuations in cAMP/cGMP concentration affect the activity of protein kinases, which – through phosphorylation – alters neuronal excitability via changes in the production, surface expression or activity of voltage or ligand-gated ion channels, including the NMDA-R itself. This mechanism is also used by another glutamatergic receptor – with pronounced modulatory effects on synaptic gain – the metabotropic glutamate receptor (mGluR; De Pasquale and Sherman, 2012). It is important to note that DA-R subtypes have opposite effects on synaptic gain: D_1_R activation stimulates cAMP production and increases the excitability of depolarized neurons, whereas D_2_R activation inhibits cAMP production and reduces gain (reviewed in Frank, 2005).

Synaptic gain is not just determined by receptor activity but also by network dynamics, like the synchronization of fast oscillations, especially in the 40–100 Hz or gamma frequencies (c.f., synchronous gain: Chawla et al., 1999). The fast acting inhibitory γ-amino butyric acid receptor (GABA_A_-R) is instrumental in this synchronization process. In the cortex, a GABAergic (parvalbumin-positive basket cell or PVBC) interneuron contacts many pyramidal cells, which it transiently hyperpolarizes. When this hyperpolarization wears off, all the cortical pyramidal cells can then fire together, leading to synchronous firing across the network and oscillations as the cycle recurs (Gonzalez-Burgos and Lewis, 2012).

Abnormalities in at least three of these synaptic gain mechanisms have been proposed to be a primary pathology in schizophrenia – those of NMDA, GABA, and dopamine receptors. NMDA-Rs play a central role in theories of schizophrenia (Olney and Farber, 1995; Abi-Saab et al., 1998; Goff and Coyle, 2001; Stephan et al., 2006; Corlett et al., 2011). Studies of genetic risk in schizophrenia have highlighted the role of genes related to glutamatergic transmission, with GABA and dopamine related genes implicated to a lesser extent (Harrison and Weinberger, 2005; Stephan et al., 2006; Hall et al., 2009; Greenwood et al., 2012). Neuropathological evidence indicates abnormalities of the glutamate and GABA systems: both pre- and post-synaptic markers, morphometric, and biochemical measures of glutamatergic transmission are reduced, as is the expression of the GABA synthesizing enzyme glutamic acid decarboxylase (GAD), parvalbumin-immunoreactive GABAergic interneurons and their synaptic markers (reviewed in Harrison et al., 2011). These neuropathological changes are particularly apparent in hippocampus and frontal cortex, both at high levels in the cortical hierarchy (Felleman and Van Essen, 1991).

Conversely, the evidence for dopaminergic abnormalities in schizophrenia is neither neuropathological nor structural, but functional. The most widely replicated abnormality is that of elevated striatal dopamine availability – in acute psychoses of both schizophrenia (Laruelle et al., 1996; Breier et al., 1997) and epilepsy (Reith et al., 1994). A recent review concluded that dopamine dysregulation is more closely linked to the state of psychosis than the trait of schizophrenia (Howes and Kapur, 2009), although there are some important caveats: presynaptic dopamine is also raised to a lesser degree in those who are prone to psychosis but not floridly psychotic, and patients with symptoms resistant to dopamine blockade do not have elevated striatal dopamine synthesis (Demjaha et al., 2012).

Is aberrant glutamatergic and GABAergic transmission linked to the trait abnormalities of the previous section? The psychotomimetic effects of ketamine suggest a strong association. Ketamine blocks NMDA-Rs and also potentiates AMPA-R signaling, leading to decreased burst firing of pyramidal neurons, with subsequent impairment of activation of GABAergic interneurons (Shi and Zhang, 2003). Ketamine administration can reproduce a whole spectrum of trait phenomena: such as SPEM abnormalities (Radant et al., 1998; Weiler et al., 2000); impaired P50 suppression (Oranje et al., 2002); diminished P300 (Gunduz-Bruce et al., 2012); reduced MMN (Umbricht et al., 2000; Schmidt et al., 2012); cognitive impairments (Kantrowitz and Javitt, 2010); and negative symptoms (Krystal et al., 1994). In fact, the only trait phenomenon that ketamine does not reproduce is a reduced susceptibility to the hollow mask illusion (Passie et al., 2003). This is in contrast to dopaminergic agonists, which do not reproduce perceptual, SPEM (Reilly et al., 2008), P50 (Oranje et al., 2004) or MMN (Leung et al., 2007) abnormalities – and have only small effects on the P300 (Luthringer et al., 1999). Indeed, prefrontal D_1_R hypoactivity has been associated with cognitive deficits and negative symptoms in animal models (Goldman-Rakic et al., 2004).

**Figure 2:**
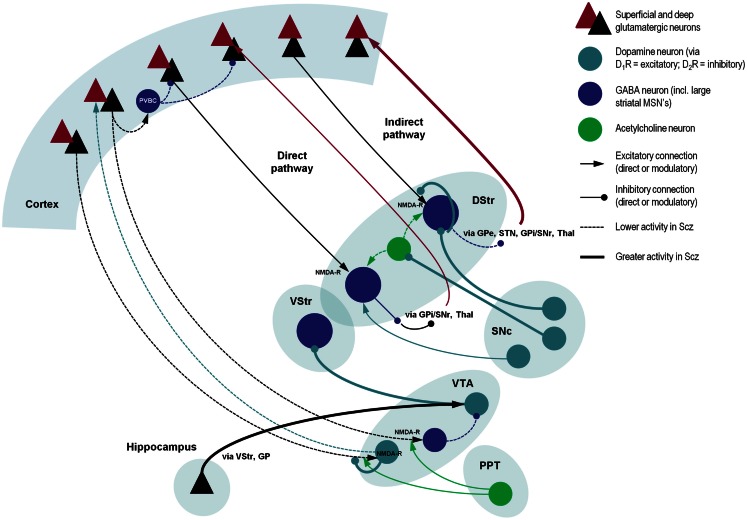
**A schematic illustration of putative pathological processes in schizophrenia – emphasizing the interactions among neuromodulatory mechanisms**. These mechanisms include: (i) decreased prefrontal NMDA-R function that may reduce the stimulation of VTA-DA neurons that project back to prefrontal D_1_Rs (decreasing cortical precision), and disinhibition of VTA-DA neurons that project to the striatum; (ii) increased dopamine release from SNc-DA neurons disinhibits the indirect pathway (by direct inhibition of striatal GABA neurons, inhibition of striatal cholinergic interneurons, and reduction of glutamate release in corticostriatal neurons); (iii) reduced NMDA-R stimulation of cortical PVBC’s reduces activity of these GABAergic interneurons, impairing coordination of cortical oscillatory activity; and (iv) increased hippocampal drive to the VTA, leading to hyperdopaminergia in the VStr. Significant omissions (for clarity) include: the GP, SNr, STN, and Thal, most connections of the VStr including its direct and indirect pathways and excitatory connections from the VTA (via D_1_Rs), and circuitry within the VStr, two more inhibitory connections in the indirect pathway and both somatic and axonal dopamine neuron D_2_ autoreceptors in SNc. As in other figures, descending projections are in black and ascending projections in red. Abbreviations: PPT, pedunculopontine tegmental nucleus; VTA, ventral tegmental area; VStr, ventral striatum; DStr, dorsal striatum; SNc/r, substantia nigra pars compacta/reticulata; GP, globus pallidus; Thal, thalamus; STN, subthalamic nucleus; PVBC, parvalbumin-positive basket cell. Stephan et al. (2009), Morrison (2012), Carlsson et al. (1999), Lisman et al. (2008), Simpson et al. (2010).

Ketamine’s reproduction of state symptoms is less consistent: its effects include loss of perceptual organization (Uhlhaas et al., 2007) and induction of a delusional mood (Corlett et al., 2011), but it does not cause a loss of attenuation of self-induced sensations (PC Fletcher, personal communication) or lead to auditory verbal hallucinations. It is interesting to note that while the negative symptoms induced by ketamine are correlated with its NMDA-R binding, the positive symptoms are not (Stone et al., 2008). Conversely, D_2_R levels in cortical and striatal areas correlate with positive but not negative symptom scores (Kessler et al., 2009). Nevertheless, some trait-like phenomena can be reproduced by both ketamine and dopaminergic agonists, such as reduced latent inhibition (Young et al., 2005; Razoux et al., 2007), blocking (O’Tuathaigh et al., 2003; Freeman et al., 2013), and the body ownership illusion (Albrecht et al., 2011; Morgan et al., 2011). This is not surprising, as there are complex interactions between glutamatergic, GABAergic, and dopaminergic neurotransmission, within and between the brain stem, striatum, and prefrontal cortex (see Figure 2). For example, hypofunction of NMDA-Rs in cortical projections to the ventral tegmental area (which are themselves regulated by D_2_ autoreceptors, nACh-Rs, and 5-HT-Rs) are in a position to reduce the activity of mesofrontal D_1_R-projecting dopaminergic neurons (that potentiate prefrontal NMDA-Rs) and increase activity (via decreased GABAergic inhibition) of mesostriatal D_2_R-projecting neurons (Stephan et al., 2009). NMDA-Rs and D_1_Rs within the same cell potentiate each other in numerous ways (Cepeda and Levine, 2006). In the prefrontal cortex, NMDA-R impairments may lead to hypofunction of GABAergic PVBC’s, disinhibition of pyramidal cells, and reduced prefrontal gamma activity (Gonzalez-Burgos and Lewis, 2012). Alternatively, NMDA-R hypofunction could impact directly on the excitability of prefrontal pyramidal cells.

The neuropathology of schizophrenia is usually associated with higher cortical systems; e.g., prefrontal cortex and the medial temporal lobe. For example, perceptual deficits in schizophrenics (and normal subjects) have been shown to correlate with frontal and temporal volume loss (Dazzan et al., 2006). The hierarchical level of a cortical area is defined in terms of extrinsic (ascending and descending) connections that have a laminar specificity: ascending (extrinsic) projections target the granular layer 4, which sends forward (intrinsic) connections to (supragranular) layers 2 and 3. These then either send further forward (extrinsic) projections up to the next hierarchical level, or pass signals down via (infragranular) layers 5 and 6 to the level below. See Bastos et al. (2012) for a review of this canonical circuitry from the point of view of predictive coding. In prefrontal cortex – as in the rest of the cerebrum – NMDA-Rs are distributed throughout the cortical layers but are most concentrated in superficial layers 2 and 3 (Jansen et al., 1989), as are D_1_Rs (Lidow et al., 1991). By contrast, D_2_Rs are much less prevalent than D_1_Rs in the cortex (by an order of magnitude) and their peak concentration is in layer 5 (Lidow et al., 1991). Nevertheless, Opris et al. (2012) have recently shown in primates that cocaine (which increases dopaminergic transmission) reduces the activity of superficial pyramidal cells (perhaps via D_2_Rs) and thereby their synchronization with layer 5 pyramidal cells in the same minicolumn – impairing performance in a working memory task.

Many of the neuropathological changes in schizophrenia are found in supragranular layers 2 and 3, with additional abnormalities in layer 5: see Harrison et al. (2011) for a fuller treatment of this complex and sometimes inconsistent literature. In brief, the somal volume of layer 3 DLPFC pyramidal cells has been found to be reduced (Rajkowska et al., 1998; Pierri et al., 2001), and these neurons have smaller basal dendrites (Glantz and Lewis, 2000; Kalus et al., 2000) and lower dendritic spine density (Kolluri et al., 2005). These changes may be caused by the neurotrophic effects of reduced NMDA-R inputs (Rajan and Cline, 1998; Monfils and Teskey, 2004) and a loss of synaptic connectivity (Perrone-Bizzozero et al., 1996; Glantz and Lewis, 1997) – perhaps with the thalamus (Lewis et al., 2001) or association cortex (Sweet et al., 2007). Others have found losses of interneurons in layer 2 in both prefrontal and cingulate cortex (Benes et al., 1991) and smaller dendritic fields of prefrontal layer 5 pyramidal cells (Black et al., 2004). In the medial temporal lobe, most abnormalities are again found in the superficial layers; such as atypical clustering of neurons in layer 2 of entorhinal cortex (Jakob and Beckmann, 1986; Arnold et al., 1991; Falkai et al., 2000).

### Summary

In summary, the main neuropathological abnormalities appear to be expressed in high hierarchical levels (prefrontal cortex and the medial temporal lobe), particularly in supragranular layers that contain superficial pyramidal cells. The main neuromodulatory (trait) abnormalities include the hypofunction of cortical NMDA-Rs and GABAergic neurons (and possibly D_1_Rs) – in contrast to the elevation of striatal D_2_R activity in (the state of) psychosis. In short, the neuropharmacological and neuropathological evidence points to abnormal neuromodulation of superficial pyramidal cells. This is important because – in predictive coding schemes – the post-synaptic gain of these cells encodes the precision of prediction error. The next section explains why this is the case, starting from basic principles.

## Neurobiological Implementation of Active Inference

This section introduces the theory behind inference in the brain. This normative account provides key constraints on the functional (computational) anatomy of action and perception. This allows one to understand (and simulate) inference in a principled way – that is also grounded in neuroanatomy and neurophysiology. We will use the formalism below to simulate some of the schizophrenic abnormalities reviewed above. These simulations rest on descriptions of the neuronal processes (differential equations) that underwrite inference in the brain. These equations are based on three assumptions:

- The brain minimizes the free energy of sensory inputs defined by a generative model.
- The generative model used by the brain is hierarchical, non-linear, and dynamic.
- Neuronal firing rates encode the expected state of the world, under this model.

The first assumption is the free energy principle, which leads to active inference in the embodied setting of action (Friston et al., 2010a). This provides a normative (Bayes-optimal) account of action and perception, in which both minimize a free energy bound on the (negative log) evidence for the brain’s model of the world. Free energy is a quantity from statistics that measures the quality of a model in terms of the probability that it could have generated observed outcomes. This means that minimizing free energy maximizes the Bayesian evidence for the generative model (Ballard et al., 1983; Hinton and van Camp, 1993; Dayan et al., 1995). The second assumption is motivated by noting that the world is both dynamic and non-linear and that hierarchical causal structure emerges inevitably from a separation of temporal scales (Ginzburg, 1955; Haken, 1983). The final assumption is the Laplace assumption that, in terms of neural codes, leads to the *Laplace code* that is arguably the simplest and most flexible of all neural codes (Friston, 2009).

Given these assumptions, one can simulate a whole variety of neuronal processes by specifying the particular equations that constitute the brain’s generative model. Action and perception are then specified completely by the above assumptions and can be implemented in a biologically plausible fashion. In brief, these simulations use differential equations that minimize the free energy of sensory input using a generalized gradient descent (Friston et al., 2010b).

(1) $$\begin{array}{l} \dot{\tilde{\mu }}\left(t\right)=D\tilde{\mu }\left(t\right)-\partial_{\tilde{\mu }}\left(\tilde{s},\tilde{\mu }\right)\\ \;\dot{a}\left(t\right)=-\partial_{a}F\left(\tilde{s},\tilde{\mu }\right) \end{array}$$

These coupled differential equations describe perception and action respectively. They say that neuronal activity encoding posterior expectations about (generalized) hidden states of the world $\tilde{\mu }=\left(\mu ,\;\mu \prime ,\;\mu \prime \prime ,\ldots \right)$ and action *a* reduce free energy – where free energy $F\left(\tilde{s},\tilde{\mu }\right)$ is a function of (generalized) sensory inputs $\tilde{s}=\left(s,\;s\prime ,\;s\prime \prime ,\;\ldots \right)$ and neuronal activity. The first differential equation is known as generalized predictive coding or Bayesian filtering: see also Rao and Ballard (1999). The first term is a prediction based upon a differential matrix operator 𝒟 that returns the generalized motion of expected hidden states. The second (correction) term is usually expressed as a mixture of prediction errors that ensures the changes in posterior expectations are Bayes-optimal predictions about hidden states of the world. The second differential equation says that action also minimizes free energy. The differential equations above are coupled because sensory input depends upon action, which depends upon perception through the posterior expectations. This circular dependency leads to a sampling of sensory input that is both predicted and predictable, thereby minimizing free energy and, implicitly, prediction errors.

To perform neuronal simulations under this scheme, it is only necessary to integrate or solve Eq. 1 to simulate the neuronal dynamics that encode posterior expectations and associated action. Posterior expectations depend upon the brain’s generative model of the world, which we assume has the following hierarchical form:

(2) $$\begin{array}{l} \;s=g^{\left(1\right)}\left(x^{\left(1\right)},v^{\left(1\right)}\right)+\omega_{v}^{\left(1\right)}\\ \;{\dot{x}}^{\left(1\right)}=f^{\left(1\right)}\left(x^{\left(1\right)},v^{\left(1\right)}\right)+\omega_{x}^{\left(1\right)}\\ \;\vdots \\ \;v^{\left(i-1\right)}=g^{\left(i\right)}\left(x^{\left(i\right)},v^{\left(i\right)}\right)+\omega_{v}^{\left(i\right)}\\ \;{\dot{x}}^{\left(i\right)}=f^{\left(i\right)}\left(x^{\left(i\right)},v^{\left(i\right)}\right)+\omega_{x}^{\left(i\right)}\\ \;\vdots \\ \;\omega_{x}^{\left(i\right)}∼N\left(0,\Pi_{x}^{(i)-1}\right)\\ \;\omega_{v}^{\left(i\right)}∼N\left(0,\Pi_{x}^{(i)-1}\right)\\ \;\Pi_{x}^{\left(i\right)}=\exp\left(\pi_{x}^{\left(i\right)}\left(x^{\left(i\right)},v^{\left(i\right)}\right)\right)\\ \;\Pi_{v}^{\left(i\right)}=\exp\left(\pi_{v}^{\left(i\right)}\left(x^{\left(i\right)},v^{\left(i\right)}\right)\right) \end{array}$$

This equation describes a probability density over the sensory and hidden states that generate sensory input. Here, the hidden states have been divided into hidden states and causes (*x*^(*i*)^, *v*^(*i*)^), with (*i*) denoting their level within the hierarchical model. Hidden states and causes are abstract variables that the brain uses to explain or predict sensations – like the motion of an object in the field of view. In these models, hidden causes link hierarchical levels, whereas hidden states link dynamics over time. Here (*g*^(*i*)^, *f* ^(*i*)^) are non-linear functions of hidden states and causes that generate hidden causes for the level below and – at the lowest level – sensory inputs. Random fluctuations in the motion of hidden states and causes $\left(\omega_{x}^{\left(i\right)},\;\omega_{v}^{\left(i\right)}\right)$ enter each level of the hierarchy. Gaussian assumptions about these random fluctuations make the model probabilistic. They play the role of sensory noise at the first level and induce uncertainty at higher levels. The amplitudes of these random fluctuations are quantified by their precisions $\left(\Pi_{x}^{\left(i\right)},\;\Pi_{v}^{\left(i\right)}\right)$ that may depend upon the hidden states or causes through their log precisions $\left(\pi_{x}^{\left(i\right)},\;\pi_{v}^{\left(i\right)}\right)$

### Perception and predictive coding

Given the form of the generative model Eq. 2 we can now write down the differential Eq. 1 describing neuronal dynamics in terms of (precision weighted) prediction errors on the hidden causes and states. These errors represent the difference between posterior expectations and predicted values, under the generative model (using *A* × *B*: = *A^T^B* and omitting higher-order terms):

(3) $$\begin{array}{c} {\dot{\tilde{\mu }}}_{x}^{\left(i\right)}=D{\tilde{\mu }}_{x}^{\left(i\right)}+\left(\frac{\partial {\tilde{g}}^{\left(i\right)}}{\partial {\tilde{\mu }}_{x}^{\left(i\right)}}-\frac{1}{2}{\tilde{\varepsilon }}_{v}^{\left(i\right)}\frac{\partial {\tilde{\pi }}_{v}^{\left(i\right)}}{\partial {\tilde{\mu }}_{x}^{\left(i\right)}}\right)\cdot \xi_{v}^{\left(i\right)}\\ +\left(\frac{\partial {\tilde{f}}^{\left(i\right)}}{\partial {\tilde{\mu }}_{x}^{\left(i\right)}}-\frac{1}{2}{\tilde{\varepsilon }}_{x}^{\left(i\right)}\frac{\partial {\tilde{\pi }}_{x}^{\left(i\right)}}{\partial {\tilde{\mu }}_{x}^{\left(i\right)}}\right)\cdot \xi_{x}^{\left(i\right)}\\ +\frac{\partial \;tr\left({\tilde{\pi }}_{v}^{\left(i\right)}+{\tilde{\pi }}_{x}^{\left(i\right)}\right)}{\partial {\tilde{\mu }}_{x}^{\left(i\right)}}-D^{T}\xi_{x}^{\left(i\right)}\\ {\dot{\tilde{\mu }}}_{v}^{\left(i\right)}=D{\tilde{\mu }}_{v}^{\left(i\right)}+\left(\frac{\partial {\tilde{g}}^{\left(i\right)}}{\partial {\tilde{\mu }}_{v}^{\left(i\right)}}-\frac{1}{2}{\tilde{\varepsilon }}_{v}^{\left(i\right)}\frac{\partial {\tilde{\pi }}_{v}^{\left(i\right)}}{\partial {\tilde{\mu }}_{v}^{\left(i\right)}}\right)\cdot \xi_{v}^{\left(i\right)}\\ +\left(\frac{\partial {\tilde{f}}^{\left(i\right)}}{\partial {\tilde{\mu }}_{x}^{\left(i\right)}}-\frac{1}{2}{\tilde{\varepsilon }}_{x}^{\left(i\right)}\frac{\partial {\tilde{\pi }}_{x}^{\left(i\right)}}{\partial {\tilde{\mu }}_{v}^{\left(i\right)}}\right)\cdot \xi_{x}^{\left(i\right)}\\ +\frac{\partial \;tr\left({\tilde{\pi }}_{v}^{\left(i\right)}+{\tilde{\pi }}_{x}^{\left(i\right)}\right)}{\partial {\tilde{\mu }}_{v}^{\left(i\right)}}-\xi_{v}^{\left(i+1\right)} \end{array}$$

$$\begin{array}{l} \xi_{x}^{\left(i\right)}={\tilde{\Pi }}_{x}^{\left(i\right)}{\tilde{\xi }}_{x}^{\left(i\right)}={\tilde{\Pi }}_{x}^{\left(i\right)}\left(D{\tilde{\mu }}_{x}^{\left(i\right)}-{\tilde{f}}^{\left(i\right)}\left({\tilde{\mu }}_{x}^{\left(i\right)},{\tilde{\mu }}_{v}^{\left(i\right)}\right)\right)\\ \xi_{v}^{\left(i\right)}={\tilde{\Pi }}_{v}^{\left(i\right)}{\tilde{\xi }}_{v}^{\left(i\right)}={\tilde{\Pi }}_{v}^{\left(i\right)}\left({\tilde{\mu }}_{v}^{\left(i-1\right)}-{\tilde{g}}^{\left(i\right)}\left({\tilde{\mu }}_{x}^{\left(i\right)},{\tilde{\mu }}_{v}^{\left(i\right)}\right)\right) \end{array}$$

Equation 3 can be derived by computing the free energy for the hierarchical model in Eq. 2 and inserting its gradients into Eq. 1. This produces a relatively simple update scheme, in which posterior expectations are driven by a mixture of prediction errors, where prediction errors are defined by the equations of the generative model.

**Figure 3:**
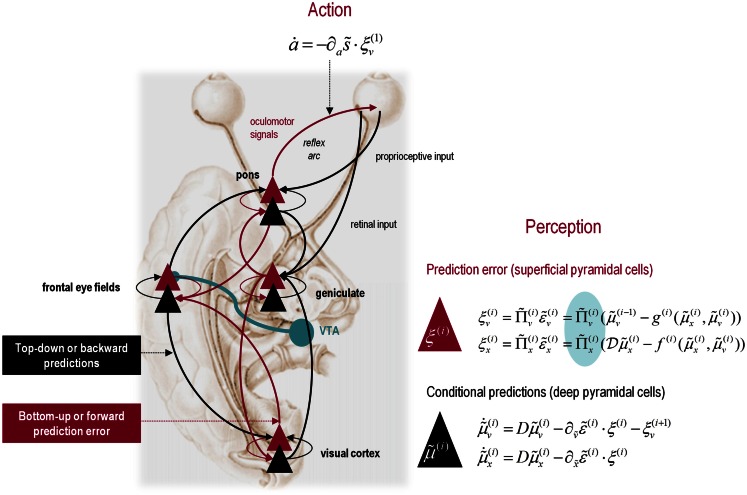
**Hierarchical message passing in the visual-oculomotor system: the schematic illustrates a neuronal message-passing scheme (generalized Bayesian filtering or predictive coding) that optimizes posterior expectations about hidden states of the world, given sensory (visual) data, and the active (oculomotor) sampling of those data**. It shows the speculative cells of origin of forward driving connections (in red) that convey prediction errors from a lower area to a higher area and the backward connections (in black) that construct predictions. These predictions try to explain away prediction error in lower levels. In this scheme, the sources of forward and backward connections are superficial (red) and deep (black) pyramidal cells respectively. The cyan connection denotes a neuromodulatory connection from the ventral tegmental area (VTA) which mediates estimates of precision. The equations on the right represent a generalized descent on free energy under the hierarchical model described in the main text – this can be regarded as a generalization of predictive coding or Bayesian (e.g., Kalman–Bucy) filtering. These equations are simplified versions of Eq. 3, in which state-dependent precision has been suppressed. State units are in black and error units are in red. The cyan circle highlights where precisions enter these equations – to modulate prediction error units (superficial pyramidal cells) such that they report precision-weighted prediction errors. In this schematic, we have placed different levels of a hierarchical model within the visual-oculomotor system. Visual input arrives in an intrinsic (retinal) frame of reference that depends on the direction of gaze. Exteroceptive input is then passed to the lateral geniculate nuclei (LGN) and to higher visual and prefrontal (e.g., frontal eye fields) areas in the form of prediction errors. Crucially, proprioceptive sensations are also predicted, creating prediction errors at the level of the cranial nerve nuclei (pons). The special aspect of these proprioceptive prediction errors is that they can be resolved in one of two ways: top-down predictions can change or the errors can be resolved through classical reflex arcs – in other words, they can elicit action to change the direction of gaze and close the visual–oculomotor loop.

It is difficult to overstate the generality of Eq. 3: its solutions grandfather nearly every known statistical estimation scheme, under parametric assumptions about additive or multiplicative noise (Friston, 2008). These range from ordinary least squares to advanced variational deconvolution schemes. The scheme is called *generalized Bayesian filtering* or *predictive coding* (Friston et al., 2010b). In neural network terms, Eq. 3 says that error units $\left(\xi_{v}^{\left(i\right)}\right)$ compute the difference between expectations at one level $\left({\tilde{\mu }}_{v}^{\left(i-1\right)}\right)$ and predictions from the level above $\left({\tilde{g}}^{\left(i\right)}\left({\tilde{\mu }}_{x}^{\left(i\right)},\;{\tilde{\mu }}_{v}^{\left(i\right)}\right)\right)$. Conversely, posterior expectations (encoded by the activity of state units) are driven by prediction errors from the same level and the level below. These constitute bottom-up and lateral messages that drive posterior expectations toward a better prediction to reduce the prediction error in the level below. This is the essence of recurrent message passing between hierarchical levels to optimize free energy or suppress prediction error: see Friston and Kiebel (2009b) and Feldman and Friston (2010) for a more detailed discussion. Crucially, in neurobiological implementations of this scheme, the sources of bottom-up prediction errors have to be superficial pyramidal cells, because it is these – and only these – cells that send forward (ascending) connections to higher cortical areas. Conversely, predictions are conveyed from deep pyramidal cells, by backward (descending) connections, to target the superficial pyramidal cells encoding prediction error (Mumford, 1992; Bastos et al., 2012): see Figure 3.

Note that the precisions depend on the expected hidden causes and states. We have proposed that this dependency mediates attention and action selection in hierarchical processing (Feldman and Friston, 2010; Friston et al., 2012). Equation 3 tells us that the (state-dependent) precisions $\left(\Pi_{x}^{\left(i\right)},\;\Pi_{v}^{\left(i\right)}\right)$ modulate the responses of prediction error units to their presynaptic inputs. This modulation depends on the posterior expectations about the states and suggests something intuitive – attention is mediated by activity-dependent modulation of the synaptic gain of principal cells that convey sensory information (prediction error) from one cortical level to the next. This translates into a top-down control of synaptic gain in principal (superficial pyramidal) cells elaborating prediction errors and fits comfortably with the modulatory effects of top-down connections in cortical hierarchies that have been associated with attention and action selection.

### Action

In active inference, posterior expectations elicit behavior by sending top-down predictions down the hierarchy that are unpacked into proprioceptive predictions at the level of the cranial nerve nuclei and spinal cord. These engage classical reflex arcs to suppress proprioceptive prediction errors and produce the predicted motor trajectory

(4) $$\dot{a}=-\frac{\partial }{\partial a}F=-\frac{\partial \tilde{s}}{\partial a}\times \xi_{v}^{1}$$

The reduction of action to classical reflexes follows because the only way that action can minimize free energy is to change sensory (proprioceptive) prediction errors by changing sensory signals; cf., the equilibrium point formulation of motor control (Feldman and Levin, 1995). In short, active inference can be regarded as equipping a generalized predictive coding scheme with classical reflex arcs: see Friston et al. (2009) and Adams et al. (2013) for details. The actual movements produced clearly depend upon top-down predictions that can have a deep and complex structure, as we will see later.

### Summary

In summary, starting with the assumption that the brain is trying to maximize the evidence for its model of the world, one can derive plausible equations describing neuronal dynamics in terms of message passing among different levels of a (cortical) hierarchical model. These messages comprise precision-weighted prediction errors that are passed forward from one level to the next and top-down predictions that are reciprocated to minimize prediction error. In this scheme, precision is encoded by the gain of superficial pyramidal cells reporting prediction error, which is implicated in the synaptic pathology of schizophrenia. This is a straightforward consequence of the mathematical form of predictive coding and the fact that superficial pyramidal cells are the source of ascending connections in the brain. At the proprioceptive level, prediction errors can be reduced either by changing predictions (perception) or by changing sensations (action). In the last three sections, we use Eqs 3 and 4 to simulate active inference under a number of generative models, while manipulating the precision at different hierarchical levels. These models are described completely by the Eq. 2, which are provided in figures that summarize the generative model used in each example.

## Perceptual Inference and Hallucinations

This section focuses on perceptual inference to show how reducing the precision at high levels of a generative model can confound perception and distort perceptual synthesis. We will examine a non-trivial problem; namely, recognizing structure and syntax in communication, using a well studied model – birdsong. This is an interesting problem because it calls upon both the dynamics modeled by hidden states and a hierarchical structure that entails a separation of temporal scales (Kiebel et al., 2009). We first describe our generative model of birdsong and then examine the sorts of inference that arise when prior precision is reduced. We then model a compensatory reduction in sensory precision. In brief, we will see a loss of responses to violations – of the sort that characterize psychotic traits (e.g., reduced MMN) – and the emergence of hallucinosis with compensatory changes in sensory precision.

### Attractors in the brain

The basic idea behind the generative model in this section is that the environment unfolds as an ordered sequence of dynamics, whose equations of motion have an attractor manifold that contains sensory trajectories. Crucially, the shape of this manifold is itself changed by other dynamical systems that have their own attracting sets. If the brain has a generative model of these hierarchically coupled dynamics, then we would expect to see cascades of neuronal attractors (c.f., central pattern generators) that are trying to predict sensory input. In this hierarchical setting, one would expect higher attractors to predict the changing shape of lower attractors, thereby modeling a separation of temporal scales of the sort seen in language (e.g., from formants to phonemes, from phonemes to words, from words to phrases, from phrases to sentences, and so on).

**Figure 4:**
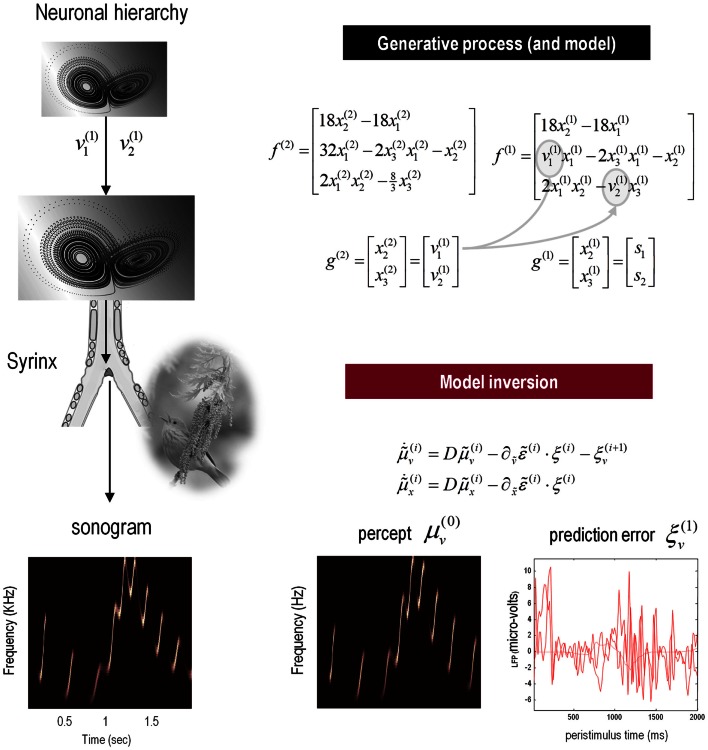
**Schematic showing the construction of the generative model for birdsongs**. This comprises two Lorenz attractors where the higher attractor delivers two control parameters (gray circles in the corresponding equations of motion) to a lower level attractor, which, in turn, delivers two control parameters to a synthetic syrinx to produce amplitude and frequency modulated stimuli. These control parameters correspond to hidden causes that have to be inferred, given the stimulus. This stimulus is represented as a sonogram (lower left panel). The upper equations represent the hierarchical dynamic model in the form of Eq. 2; while the lower equations summarize the recognition or Bayesian filtering scheme in the form of (a simplified version of) Eq. 3. The lower right panels show the sensory predictions of this Bayesian filtering scheme in terms of the predicted sonogram based upon posterior expectations (left) and the precision-weighted prediction errors driving these expectations (right).

The example used here deals with the generation and recognition of birdsongs (Laje and Mindlin, 2002). We imagine that birdsongs are produced by two time-varying control parameters that control the frequency and amplitude of vibrations of a songbird’s syrinx (see Figure 4). There has been an extensive effort using attractor models at the biomechanical level to understand the generation of birdsong; e.g., Laje et al. (2002). Here, we use attractors at higher levels to provide time-varying control over the resulting sonograms. To produce synthetic stimuli, we drove the syrinx with two states of a Lorenz attractor, one controlling the frequency (between 2 and 5 kHz) and the other controlling the amplitude or volume. The parameters of the Lorenz attractor were chosen to generate a short sequence of chirps every second or so. To endow the generative model with a hierarchical structure, we placed a second Lorenz attractor – whose dynamics were an order of magnitude slower – over the first. The states of the slower attractor entered as control parameters (the Rayleigh and Prandtl number) to control the shape of the lower attractor.

We generated a single song, corresponding roughly to a cycle of the higher attractor and then filtered the ensuing sonogram (summarized as peak amplitude and volume) using the message-passing scheme described in the previous section Eq. 3. The results are shown in Figure 4 (lower panels), in terms of the predicted sonogram and prediction error at the sensory level. These results show that – after several hundred milliseconds – the veridical hidden states and causes can be recovered and provide accurate predictions of auditory sensations. Note that the percept or predictions are not an exact copy of the stimulus – the mismatch is reflected in the prediction errors on the lower right. These prediction errors provide contextual guidance for posterior expectations about hidden causes and states. Note that prediction errors coincide with the onset of each chirp, where the prediction errors for the third chirp are more protracted – suggesting that this chirp was less easy to predict than the others.

### Omission-related responses

**Figure 5:**
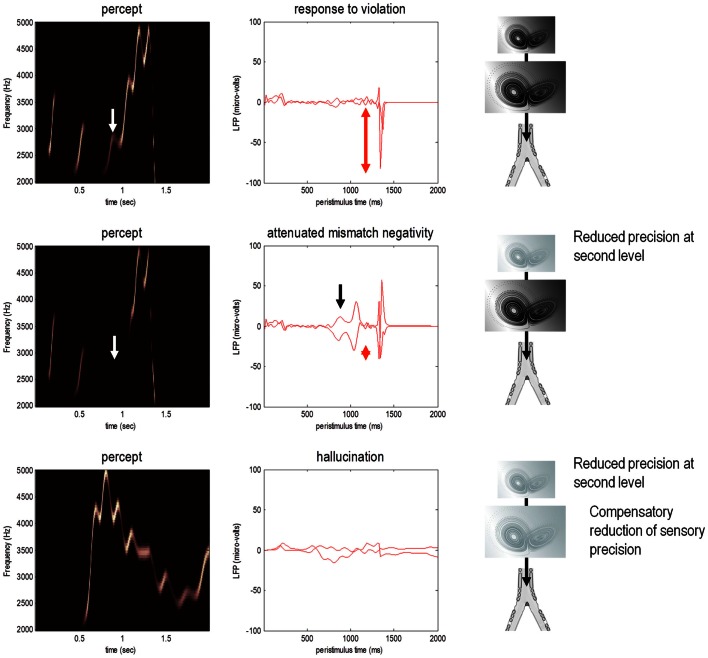
**Omission-related responses**. Here, we omitted the last three chirps from the stimulus. The left-hand panels show the predicted sonograms based upon posterior expectations, while the right-hand panels show the associated (precision weighted) prediction error at the sensory level. The top panels show a normal omission-related response using log precisions of 16 at the second (higher) level. This response is due to precise top-down predictions that are violated when the first missing chirp is not heard. This response is attenuated, when the log precision of the second level is reduced to two (middle row). This renders top-down predictions more sensitive to bottom-up sensory evidence and sensory prediction errors are resolved under reduced top-down constraints. At the same time, the third chirp – that would have been predicted on the basis of top-down (empirical) prior beliefs – is missed, leading to sensory prediction errors that nearly match the amplitude of the prediction errors elicited by the omission. The lower row shows predictions and prediction errors when there is a compensatory decrease in sensory log precision from two to minus two. Here, there is a failure of sensory prediction errors to entrain high-level expectations and subsequent false inference that persists in the absence of any stimuli.

To examine responses to surprising stimuli or violations – and how they depend upon precision – we repeated the simulation but omitted the last three chirps. The corresponding percepts are shown with their prediction errors in Figure 5 (top row). These results illustrate two important phenomena. First, there is a vigorous expression of prediction error with the first missing chirp. This reflects the dynamical nature of perception: at this point, there is no sensory input to predict and the prediction error is generated entirely by top-down predictions. Second, it can be seen that there is a transient (illusory) percept, when the missing chirp should have occurred. Its frequency is too low, but its timing is preserved in relation to the expected chirp. This is an interesting stimulation from the point of view of ERP studies of omission-related responses that provide clear evidence for the predictive capacity of the brain (e.g., Nordby et al., 1994; Yabe et al., 1997).

This simulation models neuronal responses to unpredicted or surprising stimuli of the sort used in oddball paradigms to elicit the MMN or P300. These electrophysiological markers are particularly pertinent here, because the same cells reporting prediction error (superficial pyramidal cells) are thought to be the primary source of electrophysiological measurements. In these simulations, the sensory log precision was two, the log precision of (first level) hidden states was eight and the log precision of second level prediction errors was high (16). These precisions correspond to the true uncertainty or amplitude of random fluctuations used to generate the song. So what would happen if we reduced the precision of prediction errors at the second level that provides top-down predictions about the syntax and timing of the chirps?

### Precision and oddball responses

The middle row of Figure 5 shows the results of repeating the simulation when the log precision at the second level was reduced to two. This has two remarkable effects: first, there is a failure to detect the third chirp (that previously elicited the greatest prediction error – white arrow) and, second, there is a marked attenuation of the omission–related response. The explanation for these phenomena is straightforward: because we have reduced the precision at higher levels, there is less confidence in top-down predictions and therefore every stimulus is relatively surprising. In fact, the third stimulus is so unpredictable it is not perceived, eliciting a large prediction error (black arrow in the middle right panel). Similarly, a high amplitude prediction error is seen shortly afterward in response to the surprising omission. However, it is attenuated in comparison to responses under precise top-down predictions. This allows sensory evidence to resolve prediction errors more quickly, thereby reducing their amplitude. This may speak to the attenuation of oddball responses as a psychotic trait. In particular, the attenuation of the MMN can be seen in terms of the difference between the prediction errors to the omitted chirp, relative to the third (standard) chirp (red arrows). These simulations highlight an important but intuitive point: attenuated mismatch or violation responses in chronic schizophrenia may not reflect a failure to detect surprising events but reflect a failure to detect unsurprising (predictable) events. In other words, they may reflect the fact that *every event is surprising*. In summary, a reduced precision of (confidence in) top-down predictions means that everything is mildly surprising and may provide an explanation for the failure to confidently infer regularities in the sensorium (and for larger P50 responses to repeated stimuli). As noted above, abnormal P50, P300, and MMN responses have also been demonstrated in first-degree relatives, and do not normalize with anti-dopaminergic treatment (Winterer and McCarley, 2011) – consistent with their status as trait phenomena. So what would happen if we tried to compensate for reduced prior precision by reducing sensory precision?

### Precision and hallucinations

The lower row of Figure 5 shows the results of the simulation with a compensatory reduction in sensory log precision from two to minus two. Here, the omission–related response is abolished; however there is a complete failure of perceptual inference, during the song and after its termination. Although the tempo of the percept is roughly the same as the stimulus, there is loss of frequency tracking and syntax. This false percept emerges because sensory information is not afforded the precision needed to constrain or entrain top-down predictions. The structured and autonomous nature of these predictions is an inevitable consequence of a generative model with deep structure – that is required to explain the dynamic and non-linear way in which our sensations are caused. The ensuing false inference can be associated with hallucinosis in the sense that there is a perceptual inference in the absence of sensory evidence. Clearly, the computational anatomy of hallucinations in the psychotic state is probably much more complicated – and specific to the domain of self-made acts (such as speech and movement). We will turn to the misattribution of agency in the final section. Here, it is sufficient to note that a compensatory reduction of sensory precision could produce hallucinosis of the sort seen in organic psychosyndromes. Note that the prediction error persists throughout the stimulus train and has, paradoxically, lower amplitude than in the previous simulations. This is because the prediction error is precision weighted – and we have reduced its precision.

### Summary

In summary, we have used a fairly sophisticated generative model with dynamical and hierarchical structure to recognize sequences of simulated chirps in birdsong. This is a difficult Bayesian filtering problem that the brain seems to solve with ease. The key thing to take from these simulations is that some of the trait abnormalities associated with psychosis (schizophrenia) can be explained by a loss of precise top-down predictions – rendering everything relatively surprising (c.f., delusional mood), and reducing the difference between responses to standard and oddball stimuli. The loss of precise top-down (empirical) priors can also be invoked to explain a resistance to illusions (Silverstein and Keane, 2011) that depend upon prior beliefs. We will revisit this in the context of the force-matching illusion in the last section. One can compensate for relatively precise sensory prediction errors by reducing sensory precision – but at the expense of dissociating from the sensorium and false (hallucinatory) inference. This compensated state could be a metaphor for some psychotic states. Having said this, the fact that the hallucinations of schizophrenia respond to antipsychotics suggests that they are associated with a hyper-dopaminergic state and may involve a failure of sensory attenuation of corollary discharge (see last section). In the next section, we ask what would happen if perceptual deficits of this sort occurred during active inference and affected motor behavior.

## Abnormalities of Smooth Pursuit Under Visual Occlusion

**Figure 6:**
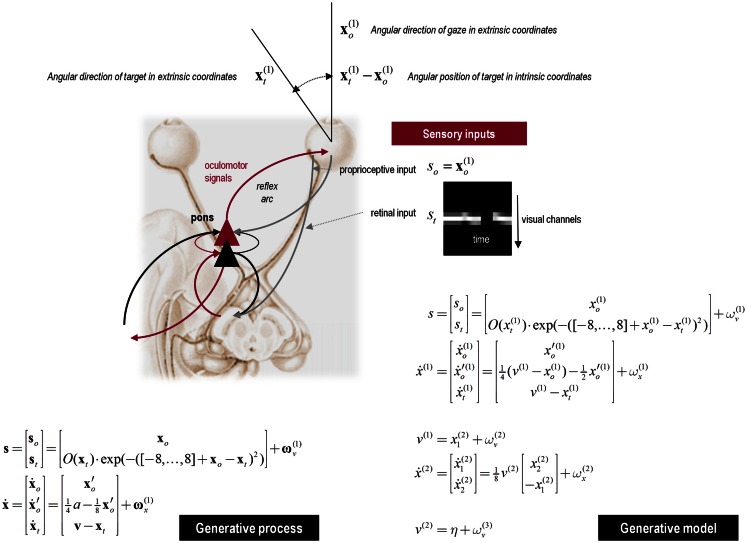
***Upper panel*: this schematic summarizes the generative model for smooth pursuit eye movements**. The model is based upon the prior belief that the center of gaze and target are attracted to a common (fictive) attracting point in visual space. The process generating sensory inputs is much simpler and is summarized by the equations specifying the generative process (lower left). The real-world provides sensory input in two modalities: proprioceptive input from cranial nerve nuclei reports the (horizontal) angular displacement of the eye **s***_o_* and corresponds to the center of gaze in extrinsic coordinates **x***_o_*. Exteroceptive (retinal) input reports the angular position of a target in a retinal (intrinsic) frame of reference **s***_t_*. This input models the response of 17 visual channels, each equipped with a Gaussian receptive field deployed at intervals of one angular unit – about 2° of visual angle. This input can be occluded by a function of target location *O*(**x***_t_*), which returns values between zero and one, such that whenever the target location **x***_t_* is behind the occluder retinal input is zero. The response of each visual channel depends upon the distance of the target from the center of gaze. This is just the difference between the oculomotor angle and target location. The hidden states of this model comprise the oculomotor states – oculomotor angle and velocity $\left({\text{x}}_{o},\;\text{x}\prime_{o}\right)$ and the target location. Oculomotor velocity is driven by action and decays to zero with a time constant of eight time bins or 8 × 16 = 128 ms. This means the action applies forces to the oculomotor plant, which responds with a degree of viscosity. The target location is perturbed by the hidden cause **v** that describes the location to which the target is drawn (a sinusoid), with a time constant of one time bin or 16 ms. The random fluctuations on sensory input and the motion of hidden states had a log precision of 16. The generative model (lower right) has a similar form to the generative process but with two important exceptions: there is no action and the motion of the hidden oculomotor states is driven by the same hidden cause that moves the target. In other words, the agent believes that its gaze is attracted to the same fictive point in visual space that is attracting the target. Second, the generative model is equipped with a deeper (hierarchical) structure that can represent periodic trajectories in the hidden cause of target motion: hidden causes are informed by the dynamics of hidden states at a second level ${\dot{x}}^{\left(2\right)}$. These model sinusoidal fluctuations of any amplitude and a frequency – that is determined by a second level hidden cause *v*^(2)^ with a prior expectation of η. This prior expectation corresponds to beliefs about the frequency of periodic motion. The log precisions on the random fluctuations in the generative model were three at the first (sensory) level and minus one at the higher level, unless stated otherwise.

This section uses a generative model for smooth oculomotor pursuit to illustrate the soft neurological signs that result from changing the precision of prediction errors in active inference. This example is particularly pertinent to schizophrenia where, arguably, some of the most reproducible signs are found in terms of eye movements. To simulate anticipatory smooth pursuit eye movements, we require a hierarchical model that generates hidden motion. One such model is summarized in Figure 6 (see figure legend for details). In brief, this model produces smooth pursuit eye movements because it embodies prior beliefs that gaze $x_{o}^{\left(1\right)}$ and the target $x_{t}^{\left(1\right)}$ are attracted by the same invisible point *v*^(1)^ in the visual field. Target motion then provides evidence that the attracting (invisible) point is moving, which induces posterior beliefs that the eye will be attracted to that moving point. These posterior beliefs create proprioceptive predictions that descend to the oculomotor system, where they are fulfilled by oculomotor reflexes (see Figure 6). Crucially, we also equipped the subject with (veridical) prior beliefs that the invisible point moves with sinusoidal motion (equations at the second level in Figure 6) – so that, during periods of visual occlusion, the subject can anticipate where the target will reappear. This part of the model constitutes the highest hierarchical level and allowed us to simulate smooth pursuit of a target with sinusoidal motion that passes temporarily behind a visual occluder.

### Simulating psychopathology

We modeled a putative deficit in schizophrenia by reducing the precision on the prediction errors of hidden states at the second level. Lowering this precision (the precision of $\omega_{x}^{\left(2\right)}$ in Figure 6) reduces the contribution of prediction errors to the posterior expectations modeling (hidden) periodic motion of the target. This results in a slowing of the (prior beliefs about the) target trajectory, as confidence in the prediction errors about its motion falls. This would normally place more emphasis on bottom-up prediction errors to guide inference; however, during occlusion these prediction errors are not available and we should see a behavioral effect of reducing precision.

**Figure 7:**
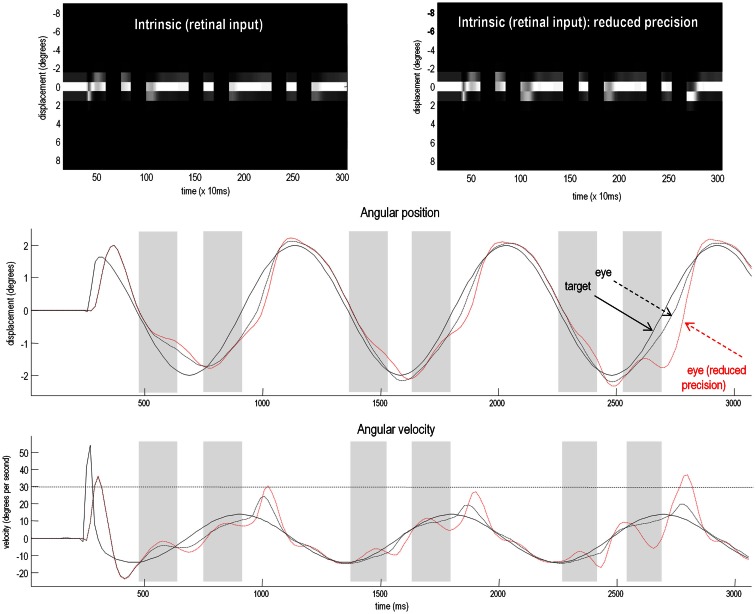
**Smooth pursuit of a partially occluded target with and without high-level precision**. These simulations show the results of applying Bayesian filtering Eq. 3 using the generative process and model of the previous figure. Notice, that in these simulations of active inference, there is no need to specify any stimuli explicitly – active sampling of the visual field means that the subject creates their own sensory inputs. The upper panels shows the responses of each of the (17) photoreceptors in image format as a function of peristimulus time. They illustrate the small fluctuations in signal that are due to imperfect pursuit and consequent retinal slip at the onset of target motion. Later, during periods of occlusion, the sensory input disappears. The lower panels show the angular displacement (top) and velocity (bottom) of the target (solid lines) and eye (broken lines) as a function of peristimulus time. They illustrate the remarkably accurate tracking behavior that is produced by prior beliefs that the center of gaze and target are drawn to the same fictive point – beliefs that action fulfils. The gray area corresponds to the period of visual occlusion. The upper right panel shows sensory input when the precision of prediction errors on the motion of hidden states at the second level was reduced from a log precision of −1 to −1.25. The associated behavior is shown with red broken lines in the lower panels. The dashed horizontal line in the lower panel corresponds to an angular velocity (30°); at which the eye movement would be considered saccadic. This simulation illustrates the loss of Bayes-optimal tracking when the motion of the target corresponds to high-level posterior beliefs but the precision of these beliefs is attenuated.

To test for these behavioral effects, we reduced the log precision on the second level from −1 to −1.25. Neurobiologically, this corresponds to a reduction in the post-synaptic gain of superficial pyramidal cells encoding prediction error in cortical areas responsible for representing regularities in target motion. Figure 7 shows the resulting active inference (upper panels) and trajectories of the target (solid black line) and eye (broken red lines) in the middle and bottom panels respectively. Comparison with the equivalent results under normal precision (broken black lines) reveals some characteristic properties of schizophrenic pursuit. First, with reduced precision, pursuit is disproportionately affected by target occlusion: at the end of occlusion, the lag behind the target is increases. This is despite the fact that when the target is visible and pursuit is stabilized, the tracking is normal (1200–1400 and 2000–2200 ms). This reproduces empirical findings in schizophrenia at modest speeds (see Thaker et al., 1999). Second, pursuit under reduced precision is less accurate on the third cycle than the first, consistent with a deficit in inferring the target trajectory. Indeed, it lags so much just prior to 2700 ms that it has to make a catch-up saccade when the target re-emerges (saccades exceed 30 °/s). Overall, these results are consistent with findings in schizophrenia that suggest an impaired ability to maintain veridical pursuit eye movements in the absence of visual information. Furthermore, they suggest that the computational mechanism that underlies this failure rests on a failure to assign precision or certainty to (empirical) prior beliefs about hidden trajectories.

**Figure 8:**
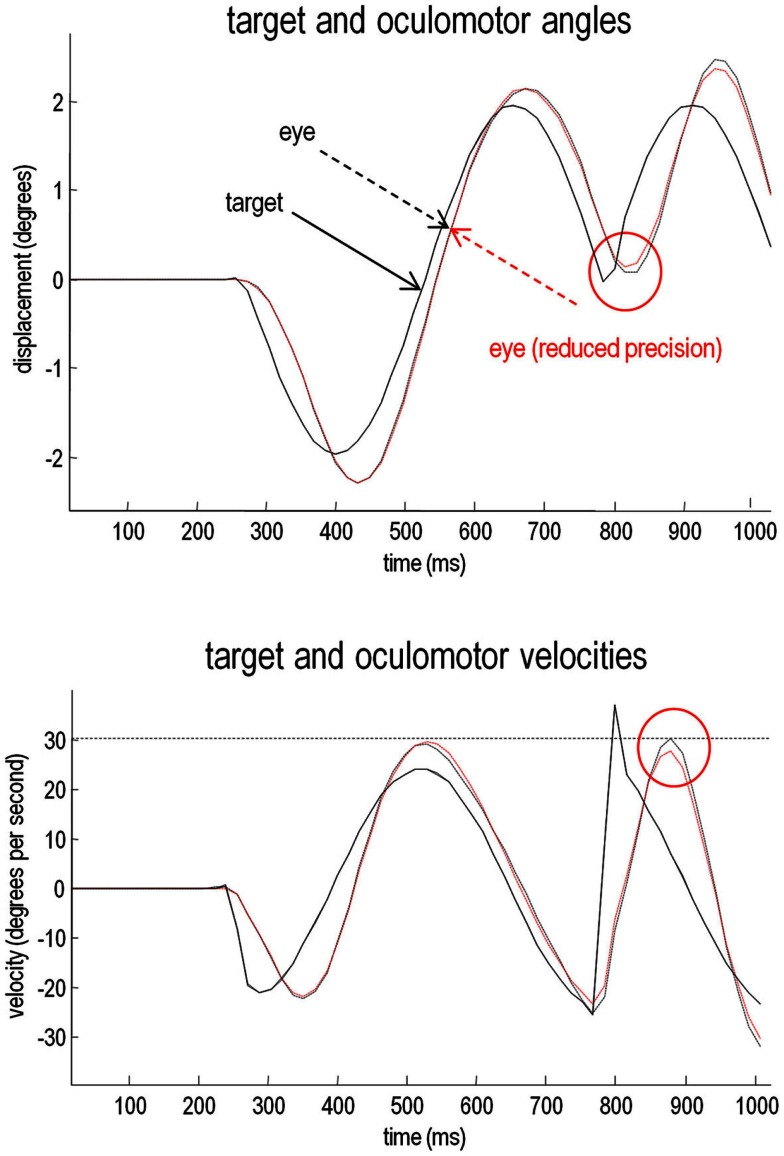
**Smooth pursuit with an unexpected trajectory change – with and without high-level precision: this figure reports the simulations of occluded periodic motion with a reversal in the direction of the trajectory at the beginning of the second cycle (plain black line)**. The broken traces in black correspond to normal pursuit and the broken traces in red show the performance under reduced precision. Although the effect is small, reducing the precision about prior beliefs produces more accurate pursuit performance, both in terms of the displacement between the target and center of gaze and in terms of a slight reduction in the peak velocity during the compensatory eye movement (red circles). This illustrates the paradoxical improvement of performance that rest upon precise sensory information that cannot be predicted *a priori* (and is characteristic of syndromes like schizophrenia and autism).

The relative loss of certainty about top-down predictions may also explain the ability of schizophrenics to respond to unpredicted changes in direction of the target. To demonstrate this, we removed the occluder, decreased the target period to around 500 ms, and introduced an unexpected reversal in the motion of the target – at the beginning of the second cycle of motion (at around 780 ms). The results of these simulations are shown in Figure 8. The traces in black correspond to normal pursuit and the traces in red show the performance under reduced precision. Although the effect is small (as it is in real subjects – Hong et al., 2005), the schizophrenic simulation (red lines) shows *more accurate* pursuit performance, both in terms of the displacement between the target and center of gaze, and in terms of a slight reduction in the peak velocity during the compensatory eye movement – a movement that is nearly fast enough to be a saccade. These differences are highlighted by red circles.

### Summary

In summary, a reduction in the precision of high-level prediction errors can account for both impaired smooth pursuit eye movements during occlusion and the paradoxical improvement of responses to unpredictable changes in target direction. This dissociation makes perfect sense from the point of view of the computational anatomy we have modeled here – reducing synaptic gain (precision) at high levels of a hierarchical predictive coding scheme reduces confidence in predictions that impairs performance when these predictions are needed (during occlusion) and that improves performance when they are not (during unpredicted motion). In the final simulations, we retain a focus on active inference but instead of attenuating high-level precision we examine the effects of failing to attenuate low-level sensory precision.

## Sensory Attenuation, Attribution of Agency, and Delusions

This section uses a generative model of (somatosensory) sensations that could be generated internally or externally. This model is used to illustrate the perceptual consequences of sensory attenuation, in terms of estimating the magnitude of externally and internally generated events. In brief, we reproduce the force-matching illusion (Shergill et al., 2003, 2005) by yoking externally applied forces to the perceived level of self-generated forces. Finally, we demonstrate the disappearance of the illusion and the emergence of false inferences about (antagonistic) external forces, when there is a failure to attenuate sensory precision and a compensatory increase in the precision of empirical prior beliefs.

### Active inference and sensory attenuation

Sensory attenuation refers to a decrease in the intensity of a perceived stimulus when it is self generated (Blakemore et al., 1998). We have suggested that sensory attenuation is necessary to allow reflex arcs to operate (Brown et al., in press). The argument is simple: proprioceptive prediction errors can only be resolved by moving – via motor reflexes – or by changing predictions. This means the effects of ascending prediction errors on posterior expectations must be attenuated to allow movement: if proprioceptive sensations are conveyed by ascending primary (Ia and Ib) sensory afferents with too much precision, then they would subvert descending predictions that create prediction errors and therefore prevent movement. It is therefore necessary to temporarily suspend the precision of sensory reafference to permit movement. If we associate the perceived intensity or detectability of the sensory consequences of action with a lower bound on their posterior confidence interval, attenuation of sensory precision provides a simple explanation for the attenuation of the perceived intensity of self-generated sensations. In what follows, we present simulations of sensory attenuation by simulating the force-match illusion and then demonstrate how overly precise prior beliefs can compensate for a failure of sensory attenuation but expose the actor to somatic delusions.

### The generative process and model

**Figure 9:**
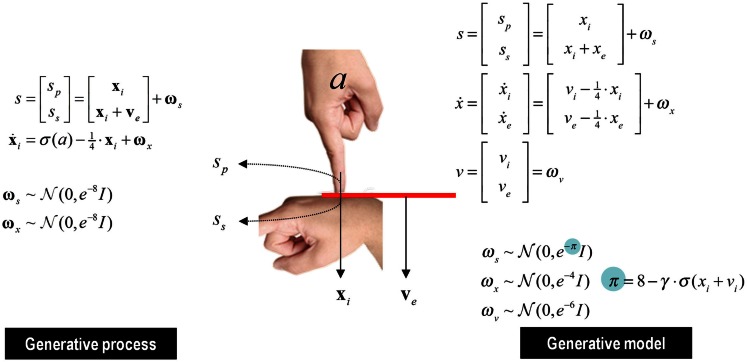
**This figure shows the generative process and model used in the simulations of sensory attenuation**. The generative process (on the left) models real-world states and causes, while the model on the right is the generative model used by the subject. In the real world, the hidden state *x_i_* corresponds to self-generated pressures that are sensed by both somatosensory *s_s_* and proprioceptive *s_p_* input channels. External forces are modeled with the hidden cause *v_e_* and are sensed only by the somatosensory channel. Action causes the self-generated force *x_i_* to increase and is modified by a sigmoid squashing function σ. The hidden state decays slowly over four time bins. In the generative model, causes of sensory data are divided into internal *v_i_* and external causes *v_e_*. The hidden cause excites dynamics in hidden states *x_i_* and *x_e_*, which decay slowly. Internal force is perceived by both proprioceptive and somatosensory receptors, as before, while external force is perceived only by somatosensory receptors. Crucially, the precision of the sensory input ω*_s_* is influenced by the level of internal force, again modulated by a squashing function, and controlled by a parameter γ that governs the level of attenuation of precision. The generalized predictive coding scheme associated with this generative model is shown schematically in the next figure.

Figure 9 summarizes the generative process and model (using the form of Eq. 2). This model is as simple as we could make it, while retaining the key ingredients that are required to demonstrate inference about or attribution of agency. The equations on the left describe the real world, while the equations on the right constitute the subject’s generative model. In the real world, there is one hidden state **x***_i_* modeling self-generated force that is registered by both proprioceptive *s_p_* and somatosensory *s_s_* inputs. Externally generated forces **v***_e_* are added to internally generated forces to provide somatosensory input. The key thing about this model is that somatosensory sensations are caused ambiguously, by either internally or externally generated forces: *s_s_* = **x***_i_* + **v***_e_*. The only way that the underlying cause of the sensations can be inferred is by reference to proprioceptive input – that is only generated internally. This is a very simple model, where the somatosensory input is used metaphorically to represent the sensory consequences of events that could be caused by self or others, while proprioceptive input represents signals that can only be caused by self-made acts. Active inference now compels the subject to infer the causes of its sensations.

**Figure 10:**
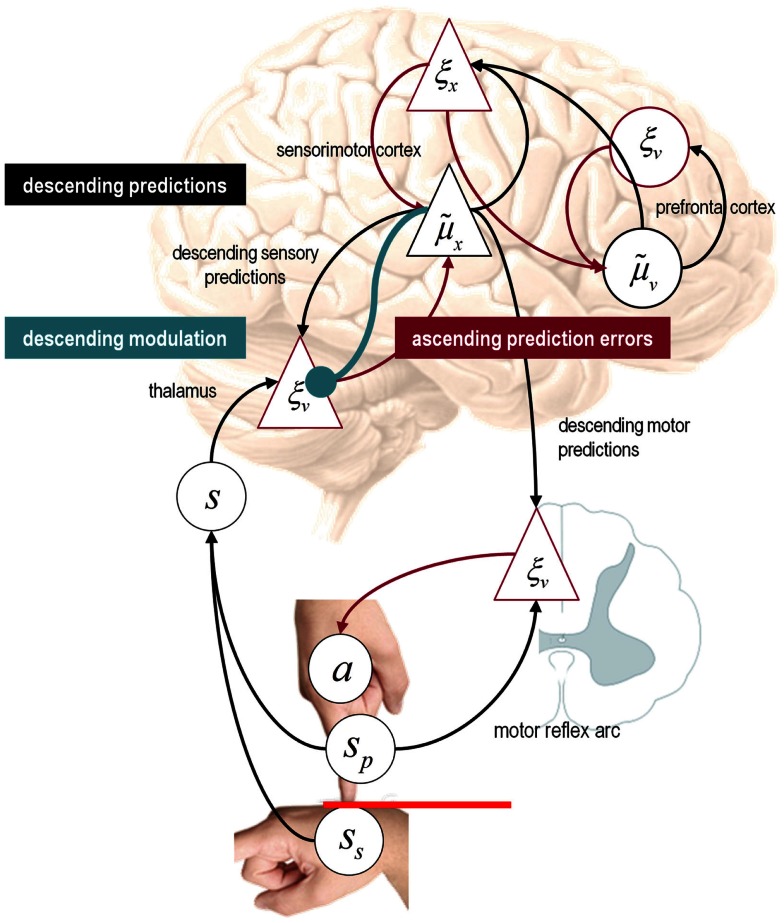
**Speculative mapping of Eq. 3 – for the generative model in the previous figure – onto neuroanatomy**. Somatosensory and proprioceptive prediction errors are generated by the thalamus, while the expectations and prediction errors about hidden states (the forces) are placed in sensorimotor cortex. The expectations and prediction errors about the hidden causes of forces have been placed in the prefrontal cortex. Under active inference, proprioceptive predictions descend to the spinal cord and elicit output from alpha motor neurons (playing the role of proprioceptive prediction error units) via a classical reflex arc. Red connections originate from prediction error units – ξ cells – and can be regarded as intrinsic connections or ascending (forward) extrinsic connections (from superficial pyramidal cells). Conversely, the black connections represent intrinsic connections and descending (backward) efferents (from deep pyramidal cells) encoding posterior expectations – $\tilde{\mu }$ cells. The cyan connection denotes descending neuromodulatory effects that mediate sensory attenuation. The crucial point to take from this schematic is that conditional expectations of sensory states (encoded in the pyramidal cell ${\tilde{\mu }}_{x}$) can either be fulfilled by descending proprioceptive predictions (that recruit classical reflex arcs) or they can be corrected by ascending sensory prediction errors. In order for descending motor efferents to prevail, the precision of the sensory prediction errors must be attenuated.

The generative model used for this inference is shown on the right. In this model, internally and externally generated forces (*x_i_*, *x_e_*) are modeled symmetrically, where changes in both are attributed to internal and external hidden causes (*v_i_*, *v_e_*). The hidden causes trigger the dynamics associated with the hidden states, much like the push that sets a swing in motion. This means that proprioceptive and somatosensory inputs are explained in terms of hidden causes, where proprioceptive sensations are caused by internally generated forces and somatosensory consequences report a mixture of internal and external forces. Crucially, the precision afforded sensory prediction errors depends upon the internally generated force (and its hidden cause). This dependency is controlled by a parameter γ that mediates the attenuation of sensory precision: as internally generated forces rise, sensory precision falls, thereby attenuating the amplitude of (precision weighted) sensory prediction errors. These context or state-dependent changes in precision enable the agent to attend to sensory input, or not – depending upon the relative precision of prediction errors at the sensory and higher levels. This context sensitive sensory precision is shown in Figure 10 as π (cyan circles).

### Functional anatomy

Figure 10 illustrates how this generative model could be transcribed into a plausible neuronal architecture. In this example, we have assigned sensory expectations and prediction errors to the thalamus, while corresponding expectations and prediction errors about hidden states (forces) are associated with the sensorimotor cortex. The expectations and prediction errors about the hidden causes of forces have been placed – somewhat agnostically – in the prefrontal cortex. Notice how proprioceptive predictions descend to the spinal cord to elicit output from alpha motor neurons (playing the role of proprioceptive prediction error units) that cause movements through a classical reflex arc. Red connections denote ascending prediction errors, black connections descending predictions (posterior expectations), and the cyan connection denotes descending neuromodulatory effects that mediate sensory attenuation. The ensuing hierarchy conforms to the functional form of the predictive coding scheme in Eq. 3. In this architecture, predictions based on expected states of the world can either be fulfilled by reflex arcs or they can be corrected by ascending sensory prediction errors. Which of these alternatives occurs depends on the relative precisions along each pathway – that are set by the descending modulatory connection to sensory prediction errors. We now use this model to demonstrate some key points.

### Sensory attenuation and the force-matching illusion

**Figure 11:**
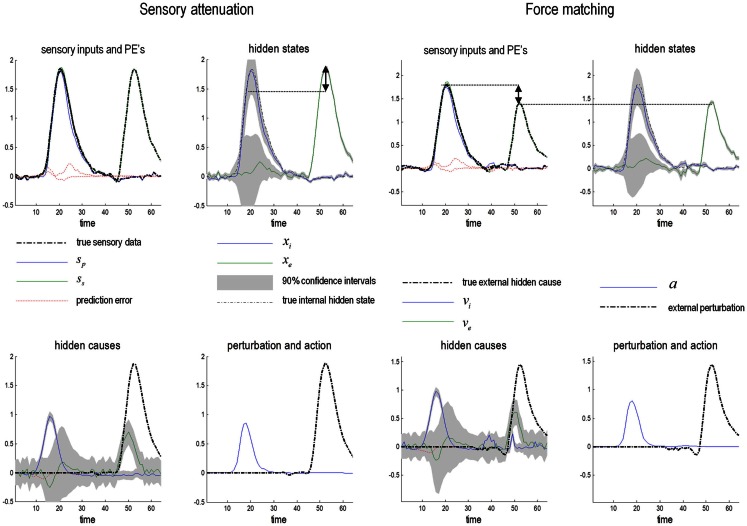
**Simulation of the force-matching task**. The *x* axes denote time in 100 ms time bins; the y axes force in Newtons. Left panels: in the first part of this simulation an internal force is generated from a prior belief about the cause *v_i_*, followed by the presentation of an external force. Posterior beliefs about the hidden states (upper right panel) are similar, but the confidence interval around the force for the internally generated state is much broader. This is because sensory level precision must be attenuated in order to allow proprioceptive predictions to be fulfilled by reflex arcs instead of being corrected by sensory input: i.e., the confidence intervals around *v_i_* must be narrower than those around *x_i_* to allow movement to proceed. If perceived intensity of the sensation is associated with the lower 90% confidence bound of the estimate of hidden state (highlighted by the dotted line), it will be lower when the force is self generated than when the force is exogenous (the difference is highlighted by the arrow). Right panels: the simulation was repeated but the external force was matched to the lower bound of the 90% confidence interval of the internal force. This means that internally generated force is now greater than the externally applied force (double-headed arrow, upper left panel). This reproduces the normal psychophysics of the force-matching illusion that can be regarded as entirely Bayes-optimal, under appropriate levels of precision.

To produce internally generated movements, we simply supplied the subject with prior beliefs that the internal hidden cause increased transiently to a value of one, with high sensory attenuation γ = 6. We then followed this self-generated movement with an exogenously generated force that matched the self-generated force. The left-hand panels in Figure 11 show the results of this simulation. The lower left panel shows the internal hidden cause (blue line) with relatively tight 90% confidence intervals (gray areas). Prior beliefs about this hidden cause excite posterior beliefs about internally generated forces, while at the same time attenuating the precision of sensory prediction errors. This is reflected by the rise in the posterior expectation of the internal force (blue line in the upper right panel) and the transient increase in the confidence interval about this expectation. The resulting proprioceptive predictions are fulfilled by action (bottom right panel) to produce the predicted sensations (upper left panel). Note that proprioceptive prediction (blue line) corresponds to somatosensory prediction (green line) and that both are close to the real values (broken black line). This simulation shows normal self-generated movement under permissive sensory attenuation.

The right-hand panels of Figure 11 show exactly the same results as in the left-hand panels; however here, we have yoked the exogenous force *x_e_* to the self-generated force *x_i_* perceived at 90% confidence (dotted line in the top right graph) – as opposed to the true force exerted by the subject. In other words, the external force corresponds to the force that would be reported by the subject to match the perceived force at 90% confidence. The 90% confidence interval was chosen as a proxy for the percept to reconcile the perceived intensity literature with results from signal detection paradigms (Cardoso-Leite et al., 2010). Experimental work in the auditory domain has demonstrated that perceived intensity can be attenuated by increasing sensory noise (decreasing precision) (Lochner and Burger, 1961; Richards, 1968). When coupled to the 90% confidence interval, the internally generated force is now much greater than the matched external force (shown on the upper left graph). This is the key finding in the force-matching illusion and is entirely consistent with sensory attenuation. In this setting, the loss of confidence in posterior estimates of hidden states that are self-generated translates into an illusory increase in the force applied, relative to the equivalent force in the absence of sensory attenuation.

**Figure 12:**
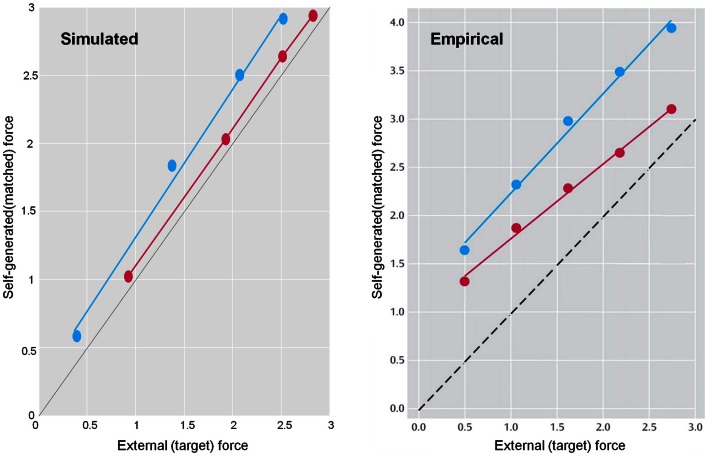
**Left panel: the force-matching simulation was repeated under different levels of self-generated force**. For normal levels of sensory attenuation (blue circles), internally produced force is higher than externally generated force at all levels. Data from patients with schizophrenia was simulated by attenuating sensory precision and increasing the precision of prediction errors at higher levels of the hierarchy. This resulted in a more veridical perception of internally generated force (red circles). Right panel: the empirical data from the force-matching task, with normal subjects’ forces in blue, and schizophrenics’ forces in red reproduced from Shergill et al. (2005).

We repeated these simulations under different levels of self-generated forces by modulating the prior beliefs about the internal hidden cause (from a half to twice the normal amplitude). The results are shown as the blue circles in the left panel of Figure 12, which plots the self-generated force against the yoked or matched external force with a corresponding 90% confidence interval. These results are remarkably similar to those obtained empirically (right panel – reproduced from Shergill et al., 2005) and reveal sensory attenuation through an illusory increase in the self-generated force, relative to matched forces over a wide range of forces. The red line in the left panel comes from the final simulations, in which we asked what would happen if subjects compensated for a failure in sensory attenuation by increasing the precision of their prior beliefs?

### False inference and failures of sensory attenuation

**Figure 13:**
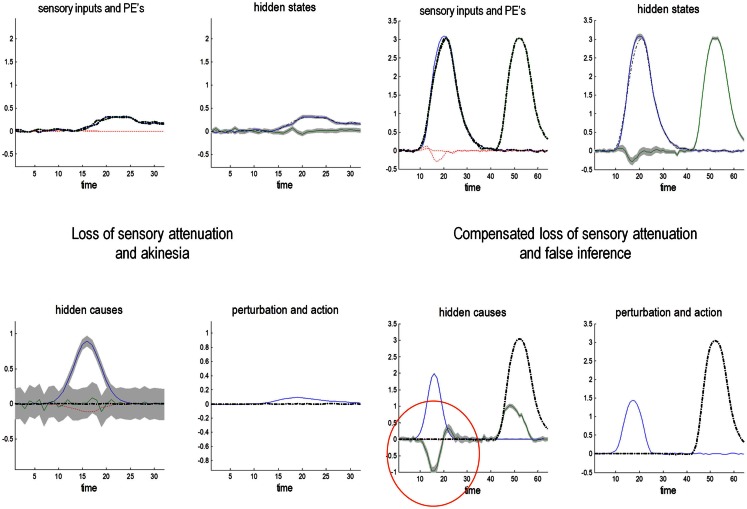
**Pathology of sensory attenuation**. Left panel: here sensory attenuation is much lower (γ = 2). In this case, bottom-up prediction errors have a higher precision than top-down predictions: the confidence intervals around *v_i_* (bottom left panel) are now broader than those around *x_i_* (upper right panel). The expected hidden state is thus profoundly suppressed (upper right panel), meaning proprioceptive prediction errors are not produced (upper left panel) and action is suppressed (lower right panel) resulting in akinesia. Right panels: to simulate the force-matching results seen in schizophrenia, precision at the second level of the hierarchy was increased to allow movement. The underlying failure of sensory attenuation still enables a precise and accurate perception of internally and externally generated sensations (upper left panel). However, the causes of sensory data are not accurately inferred: a false (delusional) cause (lower left panel) is perceived during internally generated movement that is antagonistic to the movement. This is because the proprioceptive prediction errors driving action are rendered overly precise, meaning higher levels of the hierarchy must be harnessed to explain them, resulting in a delusion that exogenous forces are opposing the expected outcome (encircled in red).

We now demonstrate two pathologies of sensory attenuation: first, a loss of sensory attenuation resulting in a catatonic state and second, how compensation for such a loss could allow movement but result in a somatic delusion. The consequences of reducing sensory attenuation (from six to two) are illustrated in the left panels of Figure 13. Here, the loss of sensory attenuation maintains the precision of the hidden states above the precision of prior beliefs about hidden causes (lower left panel). This means that bottom-up sensory prediction errors predominate over top-down predictions and expectations about internally generated forces are profoundly suppressed. Because there are no predictions about proprioceptive changes, there is a consequent akinesia. This state is reminiscent of the catatonic symptoms of schizophrenia such as immobility, mutism, catalepsy and waxy flexibility, in which the patient may maintain a fixed posture for a long time, even though (in the case of waxy flexibility) their limbs can be moved easily by someone else.

We shall now examine how a loss of sensory attenuation might be compensated for by increasing the precision of prediction errors at higher levels in the hierarchy (by increasing the log precision of prediction errors on hidden states and causes by four log units). This compensatory increase is necessary for movement and ensures the precision of top-down predictions is greater than bottom-up sensory prediction errors. These manipulations permit movement but abolish the force-matching illusion, as indicated by the line of red circles in the left panel of Figure 12. One might ask – why don’t subjects adopt this strategy and use precise prior beliefs about hidden causes all the time?

The answer is evident in the right panels of Figure 13, which show the results of a simulation with low sensory attenuation and compensatory increases in precision at higher levels. Here, there is an almost perfect and precise inference about internally and externally generated sensations. However, there is a failure of inference about their hidden causes. This can be seen on the lower left, where the subject has falsely inferred an antagonistic external hidden cause that mirrors the internal hidden causes. Note that this false inference does not occur during normal sensory attenuation (see Figure 11), where the true external hidden cause always lies within the 90% confidence intervals. The reason for this false inference or delusion is simple: action is driven by proprioceptive prediction errors that always report less force than that predicted. However, when these prediction errors are very precise they need to be explained – and can only be explained by falsely inferring an opposing exogenous force. This only occurs when both the predictions and their consequences are deemed to be very precise. This false inference could be interpreted as a delusion in the same sense that the sensory attenuation is an illusion. Having said this, it should be noted that – from the point of view of the subject – its inferences are Bayes-optimal. It is only our attribution of the inference as false that gives it an illusory or delusionary aspect.

This simulation has some face validity in relation to empirical studies of the force-matching illusion. The illusion is attenuated in normal subjects that score highly on ratings of delusional beliefs (Teufel et al., 2010). Furthermore, subjects with schizophrenia – who are prone to positive symptoms like delusions – do not show the force-matching illusion (Shergill et al., 2005). In other words, there may be a trade-off between illusions at a perceptual level and delusions at a conceptual level that is mediated by a (failure of) sensory attenuation.

### Summary

The ideas reviewed in this section suggest that attribution of agency – in an ambiguous situation – can be resolved by attenuating the precision of sensory evidence during movement: in other words, attending away from the consequences of self-made acts. When implemented in the context of active inference, this provides a Bayes-optimal explanation for sensory attenuation and attending illusions. The simulations show how exacerbations of a trait loss of sensory attenuation could subvert movement and even cause catatonia. This can be ameliorated by compensatory increases in high-level precision, which in turn necessarily induce false (delusional) inferences about agency. This is important, given the negative correlation between sensory attenuation and predisposition to delusional beliefs in normal subjects and the reduced force-matching illusion in schizophrenia. On a physiological level, increased dopaminergic transmission in the striatum could reflect a putative increase in high-level precision, compensating for hypofunction of cortical NMDA-Rs. In summary, we have shown how active inference can explain the fundamental role of sensory attenuation, and how its failure could lead to not only catatonic states but also compensatory changes that induce delusions. This is one illustration of how psychotic state abnormalities might be secondary compensations for trait abnormalities.

## Conclusion

Bayesian computations enable inference and learning under uncertainty. Furthermore, they prescribe the optimal integration of prior expectations (amassed over a lifetime or indeed evolution) with the sensory evidence of a moment; this integration is optimal because it embodies the relative uncertainty (precision) of each source of information. For this reason, the accurate representation of precision in a hierarchical Bayesian scheme is crucial for inference. The aberrant encoding of precision can therefore lead to false inference by overweighting prior expectations or sensory evidence. This paper has described how various trait abnormalities in schizophrenia could result from a decrease in prior precision (or a failure to attenuate sensory precision); and how some psychotic states could result from compensatory increases in prior precision (or decreases in sensory precision). We have outlined several physiological mechanisms for encoding precision (such as neuromodulation and neuronal oscillations) that are abnormal in schizophrenia. Genetic and neuropathological evidence suggest that NMDA-R (and GABA to some extent) may play a role in trait abnormalities, whereas the physiological evidence points toward dopaminergic pathology in the psychotic state. Clearly, a strict dichotomy is unlikely, since these neurotransmitter systems have complex interactions.

Using a biologically plausible predictive coding scheme, we have shown how a reduction of high-level (prior) precision can account for two trait phenomena: abnormal ERP responses to predictable and unpredictable stimuli and SPEM abnormalities. We have also shown how a failure to attenuate sensory precision might explain a resistance to (force-matching) illusions and (in severe cases) catatonia. Using these model systems, we were able to explain the delusional and hallucinatory inference characteristic of the psychotic state by compensatory increases (resp. decreases) in prior (resp. sensory) precision.

One might ask how specific these “trait” and “state” simulations are to schizophrenia, as opposed to psychotic symptoms *per se*. An important point to take from the formal arguments in this paper is that the common factor underlying psychotic phenomena is computational, not physiological: i.e., the key to understanding these symptoms is as disorders of precision encoding, and not – for example – necessarily of a particular neuromodulator. Another important message is that these simulations undermine a clear division between “normal” and “psychotic” brains, as even bizarre phenomena such as somatic delusions can occur in a normal inferential architecture in which precision encoding is awry. To what extent the physiological (or pharmacological) causes of transient psychotic symptoms in healthy people overlap with similar symptoms in schizophrenia is an interesting question, which physiologically informed models may help us to address (Moran et al., 2011).

Simulations of the sort used above clearly require empirical validation: this should be possible as the models make quantitative predictions about the dynamics of cortical populations that can be tested with dynamic causal modeling (Friston et al., 2003). Indeed, dynamic causal modeling studies of schizophrenic subjects have already demonstrated changes in effective connectivity consistent with decreased high level – and increased low-level – precision in the hollow mask paradigm (Dima et al., 2009, 2010). We conclude with some of the many interesting and outstanding questions in the computational modeling of schizophrenia:

- If NMDA-R and GABA transmitter systems are distributed evenly throughout the cortex, why does their pathology in schizophrenia seem to be restricted to high-level cortical areas such as the prefrontal cortex and temporal lobe? Pathology could be localized in high-level areas for genetic or developmental reasons. Alternatively, regional specificity could reflect interactions with ascending neuromodulatory projections – for example the mesocortical dopaminergic projections from the ventral tegmental area.
- Second, could the elevated presynaptic striatal dopamine in the psychotic state (Howes and Kapur, 2009) reflect (or compensate for) a primary decrease in prefrontal precision due to NMDA-R hypofunction? Or could there be another factor that reflects the contribution of developmental and environmental stressors (Giovanoli et al., 2013) or a combination of the above.
- Third, given that post-synaptic D_2_Rs reduce neuronal excitability, one might suppose that they *decrease* the precision of striatal prediction errors. The opposite may be true, however, as D_2_Rs are preferentially expressed in the indirect pathway (Figure 2), where their activation may increase cortical excitation by reducing activity in this inhibitory circuit (Fusar-Poli et al., 2011).
- Fourth, as reductionist efforts to find the best explanatory level for schizophrenia have shifted from gene-based theories to brain circuit-based accounts, a better computational understanding of trait abnormalities might enable the rational design and testing of neuromodulatory therapies; particularly those which can alleviate the debilitating antipsychotic-resistant cognitive and negative symptoms. Likewise, model-based techniques might finesse diagnostic and treatment decisions for individual patients (as has been demonstrated in aphasic patients by Brodersen et al., 2011), if the actions of different neuromodulators (for example) are formalized with appropriate models (as in Moran et al., 2011).
- Finally, schizophrenia is unlikely to be the only pathology of precision – it is notable that most current treatments for psychiatric disorders target neuromodulatory systems. Aberrant precision estimation may also prove to be a simple but powerful explanation for other psychiatric disorders; e.g., the loss of central coherence in autism (Pellicano and Burr, 2012). In future, many psychiatric disorders may be distinguished by their sites of – and causes of – variation in their encoding of precision; e.g., the precision of distributions over future action outcomes modeling helplessness in depression (Huys and Dayan, 2009).

## Conflict of Interest Statement

The authors declare that the research was conducted in the absence of any commercial or financial relationships that could be construed as a potential conflict of interest.
